# SCADIE: simultaneous estimation of cell type proportions and cell type-specific gene expressions using SCAD-based iterative estimating procedure

**DOI:** 10.1186/s13059-022-02688-w

**Published:** 2022-06-15

**Authors:** Daiwei Tang, Seyoung Park, Hongyu Zhao

**Affiliations:** 1 Department of Biostatistics, Yale School of Public Health, 60 College Street, New Haven, USA; 2 Department of Statistics, Sungkyunkwan University, 25-2, Sungkyunkwan-ro, Jongno-gu, Seoul, South Korea

**Keywords:** Deconvolution, RNA-Seq, scRNA-seq, Cell type-specific differential expression, SCAD

## Abstract

A challenge in bulk gene differential expression analysis is to differentiate changes due to cell type-specific gene expression and cell type proportions. SCADIE is an iterative algorithm that simultaneously estimates cell type-specific gene expression profiles and cell type proportions, and performs cell type-specific differential expression analysis at the group level. Through its unique penalty and objective function, SCADIE more accurately identifies cell type-specific differentially expressed genes than existing methods, including those that may be missed from single cell RNA-Seq data. SCADIE has robust performance with respect to the choice of deconvolution methods and the sources and quality of input data.

**Supplementary Information:**

The online version contains supplementary material available at (10.1186/s13059-022-02688-w).

## Background

The past three decades have seen rapid development in gene expression analysis using microarray and sequencing technologies, where bulk samples are analyzed to answer specific biological questions, e.g., to identify genes with different expression levels between cancer samples versus controls. Because bulk samples contain many distinct cell types, such analyses only provide limited granularity. Most differential expression analyses on bulk samples often assume that the measured gene expression is from the primary cell type, e.g., tumor cell in bulk tumor sample.

Recent progresses in single-cell RNA-sequencing (scRNA-seq) techniques have demonstrated substantial heterogeneity in bulk samples. However due to the high cost and complexity for scRNA-seq, most available data have remained to be from bulk samples. To make better use of bulk sample data, many in silico deconvolution methods have been proposed to infer cell type proportions from bulk data. Most deconvolution methods assume that the observed bulk gene expression profile is a convex mixture of cell-type specific gene expression profiles, i.e.,

1 $$\begin{array}{*{20}l} Y = W\cdot H,\quad W \in R^{(m\times k)+}, H \in R^{(k\times n)+},\ and\ \sum_{i=1}^{k} H_{ij}=1,\forall j. \end{array} $$

Here *Y* is the bulk gene expression matrix with *m* genes and *n* samples, *W* is the cell type-specific gene expression matrix of the *k* component cell types, and each column in *H* represents the cell type proportions for the corresponding bulk sample. All entries in these matrices are non-negative, which is indicated by the “+” sign in the notations.

The principle behind the designs of most existing deconvolution methods is to utilize genes that have distinct expression levels across cell types to infer cell type proportions. To this end, some methods curate a signature matrix $\underline {W} \in R^{m_{sub}\times k}$ with only a subset of cell type-specific genes and gather their expression profiles either from pure cell types [24, 29, 41] or scRNA-seq data [4]; others use all genes but assign higher weights to genes with more differentiating power to produce a weighted version $\tilde {\bar {W}} \in R^{m \times k}$ [42]. Both genres of methods then solve the constraint regression problem specified in the following Eqs. 2-(3) with a variety of techniques [24, 37, 40, 42].

2 $$\begin{array}{*{20}l} \underline{Y} &= \underline{W}\cdot H,\quad Y \in R^{(m_{sub} \times n)+}, \underline{W} \in R^{(m_{sub}\times k)+}, H \in R^{(k\times n)+}, \sum_{i=1}^{k} H_{ij}=1, \forall j,  \end{array} $$

3 $$\begin{array}{*{20}l} {Y} &= \tilde{\bar{W}}\cdot H,\quad Y \in R^{(m \times n)+}, \tilde{\bar{W}} \in R^{(m\times k)+}, H \in R^{(k\times n)+}, \sum_{i=1}^{k} H_{ij}=1, \forall j.  \end{array} $$

Although enormous insights on cell type proportion changes have been drawn from the applications of these deconvolution methods, most of these downstream analyses were performed under the scheme of single signature matrix, i.e., the same signature matrix was used for different groups of bulk data. In real data analyses, a more appropriate model would be that the observed differences in the bulk samples result from not only cell type compositional changes, but also from changes in cell type-specific gene expression profiles. In mathematical terms, it is *Y*_1_=*W*_1_*H*_1_ and *Y*_2_=*W*_2_*H*_2_, when an analysis is performed assuming *W*_1_=*W*_2_, it intrinsically over-attributes changes to cell type proportion changes.

In this article, we aim to simultaneously estimate group-specific *W*s and *H*s in a two-group comparison setting, thus to accurately infer cell type-specific differentially expressed genes (DEGs) as well as cell type proportion changes. To this end, we present a smoothly clipped absolute deviation-based (SCAD) iterative estimation (SCADIE) framework that can address this challenging problem.

The SCAD penalty and weighted *ℓ*_1_ penalty using the derivative of SCAD are widely used in the penalization methods [7, 21]. SCAD is defined as

$$P_{\zeta_{n}}(x) = \left\{\begin{array}{ll} \zeta_{n} |x| & \text{if}\ |x| \le \zeta_{n} \\ {\left(2a\zeta_{n} |x| -x^{2} - \zeta_{n}^{2}\right)}/{\{2(a-1)\}}\ \ \ \ \ \ \ & \text{if}\ \zeta_{n} < |x| \le a \zeta_{n} \\ {\zeta_{n}^{2} (a+1)}/{2} & \text{otherwise}, \end{array}\right. $$

where *a*,*ζ*_*n*_>0 are parameters to be tuned. SCAD can be viewed as a hybrid of *ℓ*_0_ and *ℓ*_1_ regularizers in the sense that it resembles the *ℓ*_1_ norm in a neighborhood of the origin, but stabilizes to constant at larger values [21]. Although the nonconvexity of the SCAD leads to the nonconvex optimization problem, various empirical studies have shown that it often produces estimators with smaller estimation error than the estimators via the convex *ℓ*_1_ penalty.

At high level, SCADIE is built on existing supervised deconvolution methods. It takes bulk gene expression along with a common signature matrix or initial cell type proportions as input and then estimates group specific *W*s and *H*s. Its underlying assumption is that the cell type-specific *W*_1_,*W*_2_ are reasonably similar but not exactly the same, thus it is possible to initialize with the same *W* and use an iterative algorithm to search for optimal group-specific *W*s. Through comprehensive simulation and real data analyses, we demonstrate that SCADIE is capable of identifying cell type-specific DEGs between *W*s while maintaining high accuracy in estimating *H*s.

## Results

### Overview of SCADIE

The goal of the SCADIE framework is to estimate matched *W*_1_,*H*_1_ and *W*_2_,*H*_2_ from bulk data *Y*_1_,*Y*_2_ and then perform hypothesis tests to identify cell type-specific DEGs. In the most common deconvolution scenario, only *Y*_1_,*Y*_2_ and a shared signature matrix $\underline {W}$ are provided, going from shared *W*_*sub*_ with only signature genes to group-specific *W*_1_,*W*_2_ containing all genes remains to be a challenge.

In view of this challenge, we assume the following conditions hold in our estimation setting: First, most entries in *W*_1_ and *W*_2_ are not differentially expressed. This should especially hold for signature genes in $\underline {W}_{1}$ and $\underline {W}_{2}$. In the case where systematic changes in expression profile occur across all cell types, neither should we use shared *W*_*sub*_ for initialization, nor is SCADIE applicable. Second, the compositional cell types in *W*_1_,*W*_2_ should remain the same; otherwise, different models should be used for group 1 and group 2. Common applicable scenarios include tumor microenvironments between different responding groups, or different subtypes of the same disease.

With the above assumptions, we propose a smoothly clipped absolute deviation (SCAD) penalty-based iterative estimation procedure (SCADIE) that consists of the following steps:

1. Jointly estimate cell type proportions for both groups, obtaining $H_{1}^{(0)}$ and $H_{2}^{(0)}$. Any deconvolution method can be used in this step, and by joint estimation, we assume that both groups share the same signature gene matrix $\underline {W}$ (Fig. 1 (a)). Users can also directly input $H_{1}^{(0)}$ and $H_{2}^{(0)}$ from other methods.

**Fig. 1:**
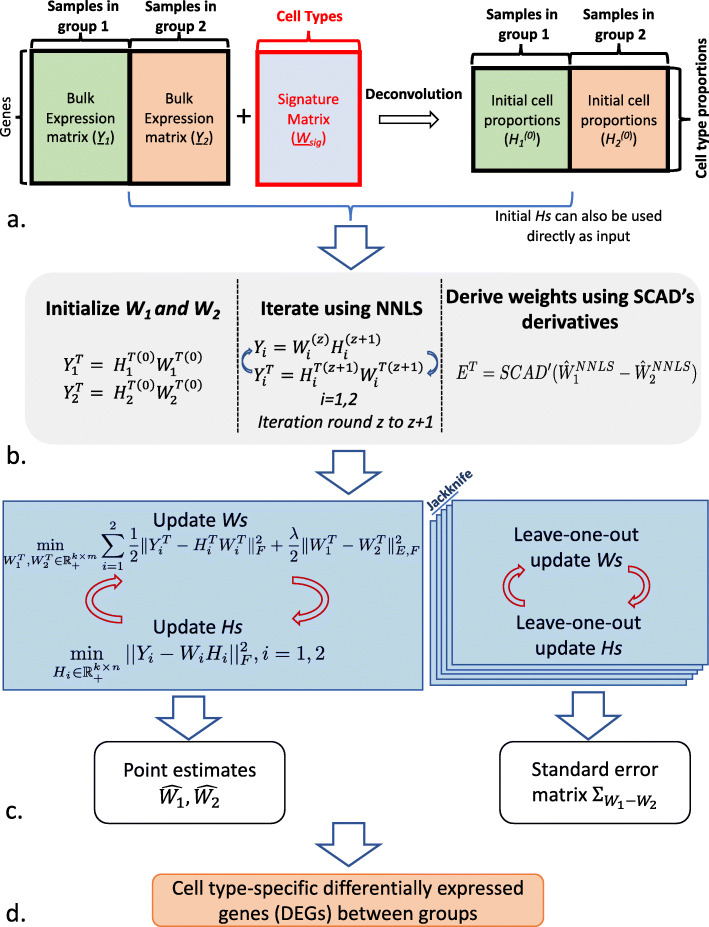
Schematic diagram for SCADIE: (a) SCADIE requires either (1) bulk gene expression matrices and cell type proportions or (2) bulk gene expression matrices and shared signature matrix as input; the cell type proportions can be obtained by any deconvolution method. (b) The initial full *W* matrices and weights are obtained by a few rounds of NNLS-based iterative updates. (c) The main iterative procedure consists of two parts: one iterative run for estimating $\hat {W}_{1}, \hat {W}_{2}$, and *n* (sample size) additional runs for leave-one-out jackknife estimation for standard error $\Sigma _{W_{1}-W_{2}}$. (d) With point estimates and entry-wise standard errors, DEGs can thus be identified
2. Obtain the initial estimates of $W_{1}^{(0)}$ and $W_{2}^{(0)}$ separately, then iteratively update *W*s and *H*s for a few rounds using non-negative least squares (NNLS).
3. Derive the weight matrix *E* used for the main estimation procedure with the derivative of SCAD function [7] (Fig. 1 (b), section “Warm-up run and weight matrix derivation”). We justify the choice of the SCAD derivative based penalty in the “Rationale behind SCAD penalty” section.
4. After completing steps 1 to 3, the main SCADIE estimation procedure consists of iteratively updating *H*_1_ and *H*_2_ using NNLS, respectively, and jointly updating *W*_1_ and *W*_2_ with a SCAD-based matrix factorization. A parallel leave-one-out jackknife procedure is also run to obtain entry-level standard error for all entries in *W*_1_−*W*_2_, and these standard errors can be summarized in a matrix $\Sigma _{W_{1}-W_{2}}$ (Fig. 1 (c) and section “Update *W* and *H*”).
5. For each pair of entries $\hat {W}^{ij}_{1}$ and $\hat {W}^{ij}_{2}$, we can calculate their *z*-score based on the standard errors of their difference $\Sigma _{W_{1}-W_{2}}^{ij}$ and then obtain a *p*-value for testing differential expression.

The above procedure outputs $\hat {H}_{1}, \hat {H}_{2},\hat {W}_{1},\hat {W}_{2}$, and $\Sigma _{W_{1}-W_{2}}$. Among these, $\hat {H}_{1}$ and $\hat {H}_{2}$ can be used for cell type proportion comparison; $\hat {W}_{1},\hat {W}_{2}$, in combination with $\Sigma _{W_{1}-W_{2}}$, can be used to perform hypothesis testing for cell type-specific differential expression analysis between the two groups.

### Simulation results

### SCADIE maintains high cell proportion estimation accuracy

One concern of SCADIE’s algorithm is that its iterative procedure and full *W*-based *H* update might result in reduced *H* estimation accuracy. To evaluate SCADIE’s performance on cell type proportion estimates, we benchmarked SCADIE against four deconvolution algorithms, including DWLS[40], CIBERSORTx[25], MuSiC[42], and a naive version of SCADIE using NNLS in updating *W*. We tested these four methods on a simulated data set, a pseudo-bulk data set[43], and a bulk microarray data with known cell type proportions[34]. We used two metrics to evaluate the accuracy of the estimated *H*s: K-L divergence and root-mean-squared error (RMSE). K-L divergence is a suitable measure because it measures the distance between two sum-to-1 discrete distributions, which are the same format as cell proportions, while RMSE is widely used by previous deconvolution methods.

Additional File 1: Supplementary Fig. S3ab shows the final output *H* accuracy compared to CIBERSORTx, DWLS, MuSiC, and NNLS-iteration, measured by K-L Divergence and RMSE, respectively. The overall result patterns between these two metrics are very consistent. In terms of performance, SCADIE showed equal or better accuracies than the other four methods except in the mouse ISC pseudo bulk dataset, where MuSiC substantially outperformed all other methods. MuSiC is specifically tailored for single cell count data, and its significantly better performance in the single cell data suggests the same. Besides, although the NNLS iteration only differs from SCADIE in its *W*-update step, the results were substantially inferior. This was due to the uncontrolled changes in *W*s over iteration and it highlights the importance of using the SCAD penalty (this will be discussed in detail in the section “Rationale behind SCAD penalty”). Further, the accuracy of the estimated *H* was stable over iterations (Additional File 1: Supplementary Fig. S3cde). Specifically, the results for the true bulk data were flat because there was only one group of samples; thus, there was no separate updating.

These results suggest that although our iterative procedure uses full *W* to update *H*, it would not negatively impact cell type proportion estimation. However, to accommodate potentially different needs, we also made signature-only updates as an option in the SCADIE package.

### SCADIE can better identify DEGs

One of SCADIE’s key features is to estimate condition-specific *W* matrices. To demonstrate the efficacy of SCADIE’s framework for this feature, we next compared its performance with four other methods with similar functions.

The most straightforward way to estimate *W* is by solving *Y*^*T*^=*H*^*T*^*W*^*T*^ using NNLS. We implemented this method in our SCADIE package and labeled it as “NNLS”. A similar technique using ordinary regression was adopted in a microarray deconvolution method called csSAM from [34]. A recently proposed statistical framework named TOAST that aims at performing hypothesis testing for cell type-specific gene expression [16] is also included. Finally, CIBERSORTx has a high resolution mode for sample-specific *W* estimate, whose results can also be used for DEG analysis.

**Fig. 2:**
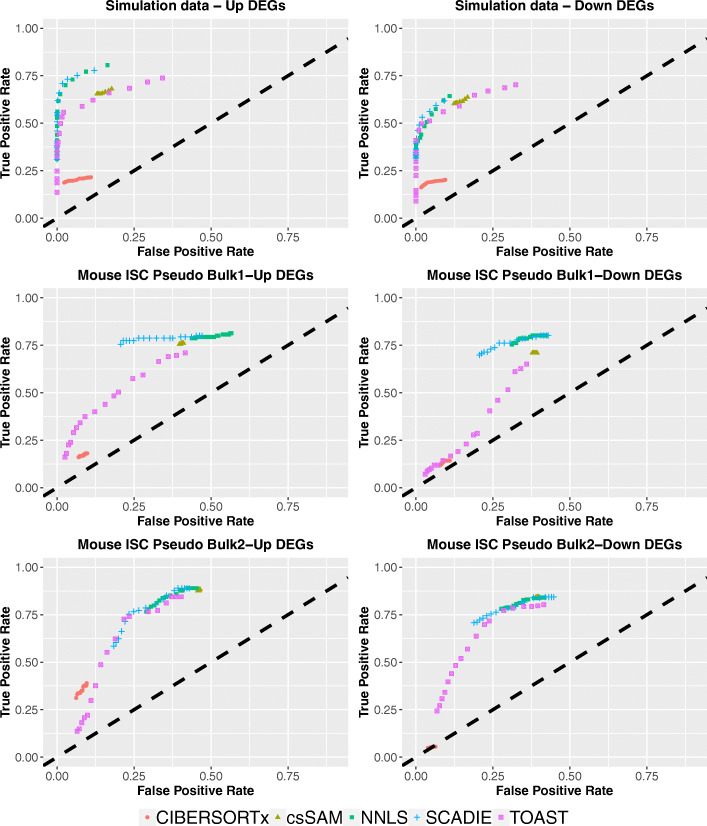
Comparison of DEG identification performances for SCADIE, NNLS *W* update, csSAM, TOAST, and CIBERSORTx’s high resolution mode under a series of *p*-value cutoff from 10^−12^ to 0.5

Here we benchmarked SCADIE against the above four methods on one simulated dataset and two pseudo-bulk datasets generated from scRNA-seq data. Because these were simulated data with known DEG statuses, we were able to measure the true-positive and false-positive rates (see the “Methods” section for more details). We plot the true-positive rates against false-positive rates over a range of *p*-values (from 10^−12^ to 0.5) for each method on each dataset in Fig. 2.

As shown in Fig. 2, SCADIE performed the best for all three datasets in identifying both up-DEGs and down-DEGs. Specifically, SCADIE outperformed CIBERSORTx, csSAM, and TOAST in both true-positive identification and false-positive control. It should be noted that since CIBERSORTx’ high resolution mode is not designed to impute group-specific gene expression levels for whole transcriptome, only a small fraction of *W*s was output from it, and the missing results for most genes were reflected in the overall low true-positive and false-positive rates. When compared to the NNLS *W*-update, SCADIE showed similar power in true-positive identification, but better false-positive controls. This is consistent with our understanding of SCAD-penalty’s advantage, which is also shown in simulation results from Fig. S2 (where false positives measured by PPV): as separate NNLS *W*-update may cause too much divergence between *W*_1_ and *W*_2_ over iterations, leading to more false positive results.

### SCADIE can improve the estimates from other methods

We next asked the question whether the initial results from CIBERSOTx and csSAM can be improved via SCADIE’s iterative procedure. To investigate this, we initialized the SCADIE algorithm with output from CIBERSORTx and csSAM on the same three datasets above and used SCADIE to iteratively estimate *W*s and *H*s, followed by DEG analysis with SCADIE’s framework. TOAST was not included in this analysis because it only performs hypothesis test without providing point estimates for *W*s.

**Fig. 3:**
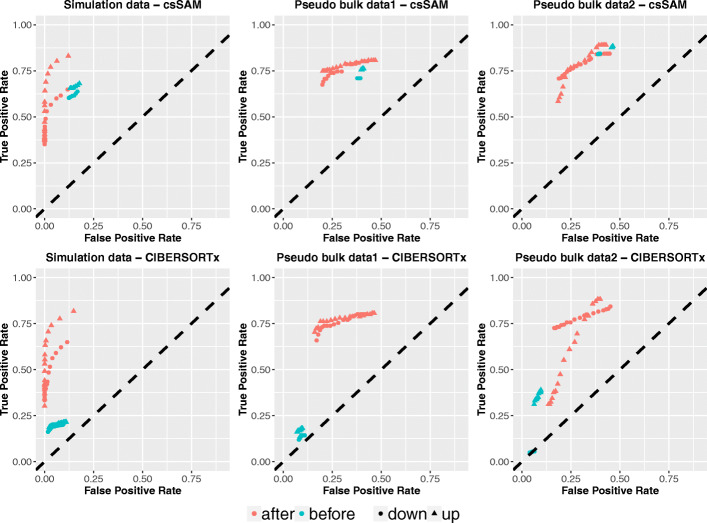
Applying SCADIE’s iterative procedure on initial estimates of CIBERSORTx and csSAM improves DEG identification accuracy

As can be seen from Fig. 3, the accuracy of DEG improved in 11 out of 12 cases (including both down- and up-DEGs) through this scheme. This demonstrates the efficacy of SCADIE’s iterative procedure in improving DEG identification accuracy.

### Robustness with respect to *ζ*_*n*_

Since the parameter *ζ*_*n*_ of the SCAD as in (6) (see the “Methods” section at the end) plays a crucial role in defining similarity penalty via SCAD, it is important to examine the robustness of SCADIE with respect to the choice of *ζ*_*n*_. To this end, we performed comprehensive sensitivity analyses on both simulation and real data for a wide range of *ζ*_*n*_ (Additional File 1: Supplementary section S3.2). The results suggest that when *ζ*_*n*_ is within a reasonable range (from 1 to 8), SCADIE’s output is highly robust in terms of *H* estimates, *W* estimates, and the DEG identifications (see Additional File 1: Supplementary section S3.2 for more details).

### SCADIE’s performance on real data

In the previous sections, we examined SCADIE’s performance extensively through simulation and pseudo-bulk data. Compared to simulated data, real datasets rarely come with ground truth cell type proportions nor cell type-specific gene expression. In addition, biopsy heterogeneity and platform/technical variation present huge uncertainty in estimation outcome.

To evaluate SCADIE’s performance on real datasets, we applied SCADIE on four bulk datasets with distinct features, from microarray to post-mortem RNA-Seq. Due to the lack of groundtruth, we primarily evaluated from the following four aspects: 1. Can SCADIE identify biologically meaningful cell type proportion changes? 2. Can SCADIE identify known cell type-specific DEGs? 3. For DEGs identified from SCADIE, are they associated with known biological processes? and 4. Can the iterative procedure improve estimation accuracy?

### SCADIE accurately infers cell type proportions and cell type-specific genes in chronic obstructive pulmonary disease

Chronic obstructive pulmonary disease (COPD) is a chronic lung disease and a leading cause of death. Many efforts have been made to profile the transcriptomes from COPD patients [1, 14, 28, 32]. To assess SCAIDE’s performance on COPD data, we derived signature matrix from a COPD single cell dataset [1] and performed deconvolution on an independent bulk data set [14] with both COPD and control samples (98 COPD samples; 91 control samples), for five major cell types (stromal, myeloid, lymphoid, epithelial, and endothelial) as clustered in [32].

**Fig. 4:**
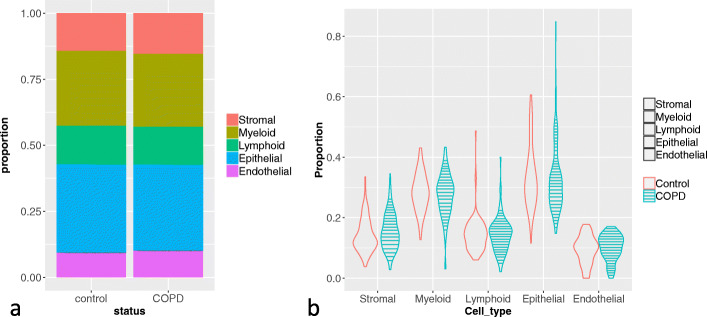
SCADIE-estimated cell proportions in control and COPD samples: **a** Mean proportions of the five cell types in control and COPD samples. **b** Boxplot for each cell type’s proportions across all individuals in the two cohorts

Although COPD causes pathological changes in several myeloid, epithelial, and endothelial cell types, previous studies did not find any systematic changes in cell type proportions associated with COPD [28, 32]. Reasons for this include the high heterogeneity of disease [26], as well as high variability in cell type compositions across specimens, which makes it difficult to identify consistent patterns. Cell type proportions estimated from SCADIE suggest similar pattern, where the mean cell type proportions are consistent between the COPD and control groups (Fig. 4a), while individual compositions varied across samples (Fig. 4b). The epithelial proportion varied most, as it is not only associated with biopsy spatial location, but also with disease severity [39].

**Fig. 5:**
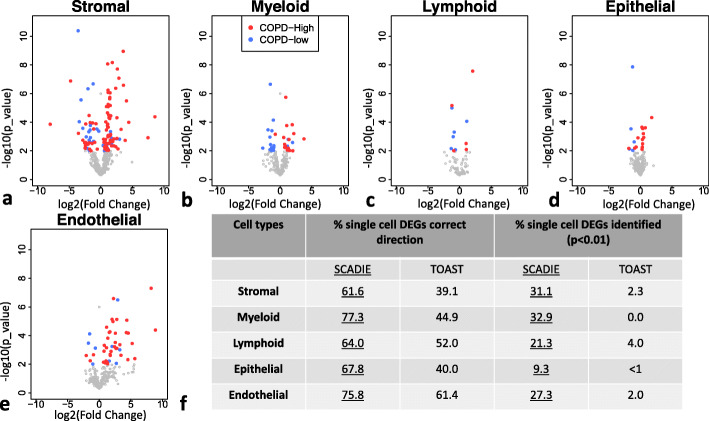
Volcano plots for single cell DEGs’ estimated differential expression statuses from SCADIE and their summary: **a**–**e** Volcano plots for single cell-identified DEGs in the five component cell types, *x*-axis represents the log2 fold-change from SCADIE, and *y*-axis represents each gene’s *p*-value from SCADIE. **f** A summary table for benchmarking SCADIE against TOAST using the same COPD datasets, the first two columns summarize the percentages of single cell DEGs that were of concordant directional changes from bulk data, and the last two columns summarize the percentages of single cell DEGs that were correctly identified as DEGs from bulk data

We next evaluated SCADIE’s performance in identifying cell type-specific DEGs from bulk data. “Ground truth” cell type-specific DEGs were first obtained by performing differential expression analysis on the COPD-Control single cell data cohort[32] for the five major cell types. Their log2 fold changes as well as *p* values are shown in volcano plots in Fig. 5a to e. Across all cell types, more than 60% of the single cell-identified DEGs showed concordant directional changes from SCADIE output. In terms of significant DEGs, 9–33% of single cell identified DEGs were also inferred to be significant DEGs by SCADIE from bulk data (Additional File 1: Supplementary Fig. S4). Since there were only fewer than half of single cell DEGs replicated in bulk data, we next asked the question of whether it was due to data heterogeneity or method deficiency. To this end, we compared SCADIE with TOAST in terms of correct direction percentage (percentage of single cell derived cell type-specific DEGs that have same directional change from SCADIE or TOAST) and correct significant DEGs percentage (percentage of single cell derived cell type-specific DEGs that are also identified significant from SCADIE or TOAST). SCADIE consistently outperformed TOAST by significant margins in both aspects (see Fig. 5f). This result suggests that there is indeed significant heterogeneity between these two unrelated single cell and bulk data cohorts, and SCADIE outperformed existing method even under this noisy circumstance. Here we only included TOAST in comparisons because csSAM only works on microarray data, while CIBERSORTx does not infer cell type-specific DEGs at the whole transcriptome level.

Note that from Supplementary Fig. S4 there is a substantial number of SCADIE-inferred DEGs that were not present in single cell DEGs; it is important to find out whether the large proportion of non-overlapping DEGs was due to false positives. To this regard, we ran Gene Set Enrichment Analysis (GSEA) on single cell and SCADIE-derived DEGs separately and compared their top enriched pathways. Results from Additional File 1: Supplementary Fig. S5 show that the top pathways from single cell and SCADIE were significantly more overlapped than that expected by chance, indicating that the underlying biological signals do share consistent patterns even the actual DEG sets differ.

Finally, we asked the question whether SCADIE can infer DEGs that were not identified in single cell data. To answer this question, we looked into the top 10 SCADIE-inferred down DEGs in each cell type. Among the 50 genes, only 8 of them were also identified from single cell DE analysis. Among the 42 DEGs unique to SCADIE, 39 were also present in the single cell dataset, and 33 showed concordant cell type-specific directional changes as SCADIE. This finding suggests that the cell type-specific expression changes from SCADIE were likely true signals, and the main reason they were missed in single cell data was the limited power due to data sparsity (See Additional File 2: Supplementary Table S1 for details). In addition, literature search showed that 19 out of the 42 SCADIE-unique genes were associated with COPD from previous studies (See Additional File 2: Supplementary Table S1 for details). This suggests that SCADIE is capable of mining DEG information that is too sparse to be identified in single cell data.

In summary, SCADIE is not only capable of identifying known cell type proportion patterns and cell type-specific DEGs, it can also infer DEGs that may be missed by single cell data due to the high noise and drop-out in single cell data.

### SCADIE reveals biologically meaningful composition and expression differences in DLBLC subtypes

Diffuse large B cell lymphoma (DLBCL) is the most common type of non-Hodgkin lymphoma and can be classified into two main subtypes based on gene expression: germinal center-like (GCB) and activated B cell-like (ABC) DLBCL [2, 19]. Traditionally DLBCL patients were treated with cyclophosphamide, doxorubicin, vincristine, and prednisone (CHOP), and in recent years CHOP in combination with immunotherapeutic drug rituximab (R-CHOP) has gained more popularity due to its benefit in clinical outcome [33].

**Fig. 6:**
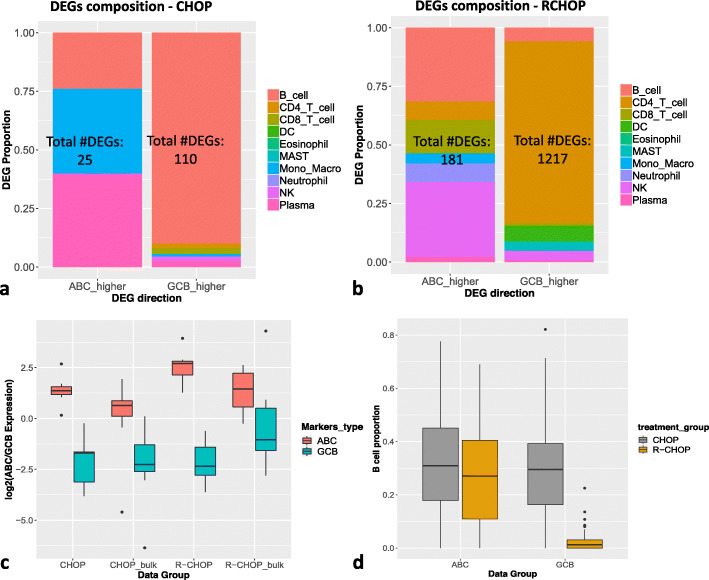
DLBLC Cell type-specific DEGs and proportion results: **a** The composition of DEGs by cell type in the CHOP cohort. **b** The composition of DEGs by cell type in the R-CHOP cohort. **c** A comparison of marker genes’ log2 fold change in estimated *W*s and original bulk data. **d** Estimated B cell proportion by disease subtype and treatment; in each case, a reduction in B cell proportion was observed

Here we analyzed a dataset consisting of 414 samples from both subtypes who received either CHOP or R-CHOP [15]. Inspired by a previous analysis from [25], we first asked the question whether the DEGs between GCB and ABC can be attributed mostly to B cells. To this regard, we applied SCADIE to all samples and compared the cell type-specific DEGs by treatment groups using the same cell type characterization and signature matrix from [25]. From Fig. 6ab, we can observe distinct DEG composition patterns between CHOP and R-CHOP patients: in the CHOP group, DEGs of GCB are dominantly by genes from B cells, and those of ABC are also from B cells or activated B cells (plasma cell), while in the R-CHOP group, there is a substantial reduction in B cell DEGs and most DEGs are instead from T cells. While this may seem counterintuitive at first, given the rituximab’s nature as an antibody against B cells, it is consistent with several lines of previous studies [18, 30] that rituximab reduces B cell proportion substantially in the GCB group (Fig. 6d) and alters T cells gene expressions more than B cells.

Next, we compiled a list of validated markers for GCB and ABC from published studies [2, 10] (see a list of all genes at [38]) and examined if SCADIE could accurately infer their distinct expressions. The log2 fold changes in the estimated B cell expressions between ABC and GCB are shown in Fig.6c, where we can see that not only all markers show higher expression levels in their corresponding cell types; their estimated fold changes in B cell are also higher compared to those in the bulk data.

Unlike previous analyses, although in real data we do not have comprehensive differential expression ground truth for all genes, we are able to demonstrate that the cell type proportions and cell type-specific DEGs inferred from SCADIE align well with the literature.

### SCADIE improves AD-associated cell type-specific DEG estimation

Alzheimer’s disease (AD) is a leading threat to global elder population and has been under intensive research over decades. However, gene expression analyses remain challenging due to the difficulty in accessing samples and the low quality of post-mortem RNA-Seq samples. To examine SCADIE’s performance on these challenging data, we applied SCADIE to an AD bulk RNA-Seq dataset where the cell type proportions had been measured by immunohistochemistry (IHC) [27]. The dataset contains RNA-Seq samples from 31 healthy individuals and 18 AD patients. We initialized *W*s with the IHC-estimated proportions and ran the iterative procedure subsequently. Due to different cell type categorizations between the IHC study [27] and DEG study [22], we only examined the three overlapped cell types: astrocyte, oligodendrocyte, and microglia (see the section “Methods—Real data processing and analyses” for details.)

**Fig. 7:**
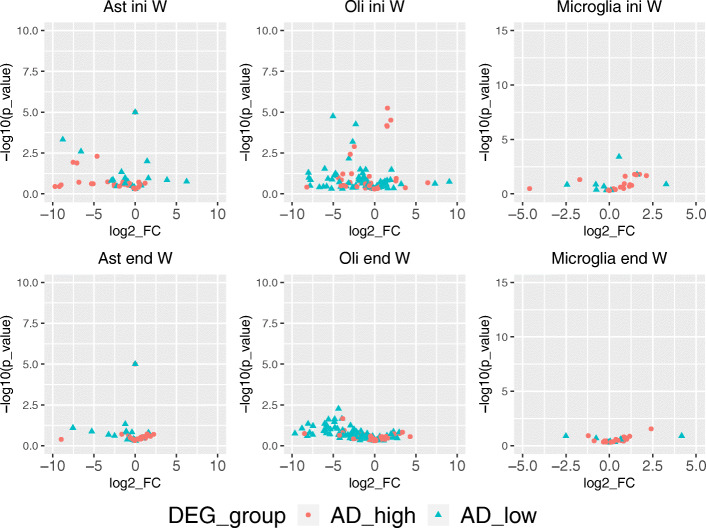
Volcano plots for cell type-specific AD-associated DEGs in initial *W*s and SCADIE output

To evaluate SCADIE’s performance, we obtained a list of cell type-specific DEGs from [22] and tested if SCADIE could correctly identify them. Figure 7 shows the estimated expression levels for those known up- and down-DEGs in the initial and final *W*s, respectively. In the initial *W*s, 34%, 20%, and 14% of known DEGs were estimated to have fold-changes in the opposite directions (i.e., up(down)-DEGs were estimated to be down(up)-regulated) with | log2*FC*|>1) in astrocyte, oligodendrocyte, and microglia. After SCADIE procedure, these ratios reduced to 5%, 9%, and 7%, respectively (Fig. 7).

These results suggest that, although low initialization quality might limit the performance of SCADIE, its iterative procedure could still improve and recover the DEGs’ directional signals.

### DEG identification under poor initialization and limited sample size

Although SCADIE can be well tuned to identify DEGs, it may run the risk of increasing error when the initial *W* and *H* deviate from ground truth. In addition, the jackknife method might perform poorly when sample size is relatively small compared to the number of cell types. To study the potential impacts of these issues, we first benchmarked SCADIE’s performances under different initial *H* accuracy levels and sample sizes. SCADIE’s accuracy decreases with both the initial *H*’s accuracy (Additional File 1: Supplementary Fig. S6) and the reduction in sample size (Additional File 1: Supplementary section S3.4), while *H* accuracy has a larger effect. This is in line with our expectation because *H* directly affects point estimates of *W*s while sample size’s effect is more on standard error.

On real data performance, we benchmarked SCADIE on a previously studied follicular lymphoma (FL) bulk dataset with both issues present. FL is a common type of B cell lymphoma and can be classified into two subtypes by the presence of CREBBP mutation [9]. We re-analyzed a cohort of 26 samples whose genotypes are known (16 CREBBP-mutant and 10 CREBBP-wild type) from [25] and benchmarked against a list of known DEGs identified by [9].

**Fig. 8:**
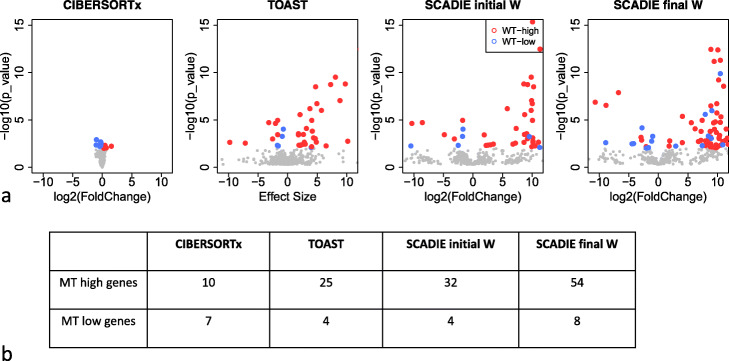
Benchmarking SCADIE on FL bulk dataset with poor initialization and limited sample size. **a** Volcano plots for known DEGs from CIBERSORTx, TOAST, SCADIE’s initial *W*s and final *W*s, for the latter two cases the standard errors were obtained through jackknife. **b** Number of correctly identified DEG (*p*-value cutoff 0.01) from CIBERSORTx HiRes, TOAST, and SCADIE. For SCADIE’s initial *W* and final *W* cases, their point estimates were different *W*s but the same set of standard deviation was used in the hypothesis tests

We inferred the initial *H* via deconvolution using CIBERSORTx’s LM22 signature matrix. Since this *H* contains 22 cell types while our sample number and leave-one-out jackknife require the number of cell type not exceeding 9, we merged *H* into eight main cell types based on their abundances and similarities. Then, the initial *W*s were obtained for CREBBP-mutant and CREBBP-wild type groups respectively. To measure the qualities of the initial *W*s, we made the volcano plot for known DEGs in initial *W*s. The results show few correctly identified down-DEGs with some false positives (Fig. 8a “SCADIE initial *W*”) in the initial *W*s. After iteration, the final *W*s correctly identified more DEGs in both directions (upregulated and downregulated, see Fig. 8a “SCADIE final *W*”). This is consistent with the table in Fig. 8b.

We next compared SCADIE with CIBERSORTx’s high resolution mode and TOAST. Since CIBERSORTx cannot perform whole-transcriptome imputation, we only input the 467 known DEGs for imputation. The results suggest that although SCADIE’s estimations had more directional errors, its overall power (Fig. 8a) and number of correctly identified DEGs (Fig. 8b) exceeded CIBERSORTx. Outcome from TOAST is very similar to SCADIE’s initial outcome (Fig. 8a), but SCADIE’s final *W*s can identify more DEGs in both directions, though at a cost of higher false positives.

The above results suggest that incorrect initialization and limited sample size do lower the estimation quality of SCADIE; however, SCADIE can still improve and maintain competitive performance in these scenarios. It is recommended to consider SCADIE’s applicability since poor initialization quality or limited sample size is common in real applications. Though it is usually not possible to evaluate initial *H*’s quality, we recommend having sample size at least 1.5x the number of cell types when performing DEG identification with SCADIE.

## Discussion

Recent years have seen rapid developments of technologies and computational methods to enable researchers to identify genes with different expression levels across conditions, either through bulk samples or single cells. When a gene is differentially expressed between two groups of bulk samples, should we attribute it to cell type proportions changes, or cell type-specific gene expression changes?

To this end, we have developed SCADIE, an estimation framework to simultaneously estimate both cell type proportions *H* and cell type-specific gene expressions *W*. SCADIE features an iterative update procedure of *W* and *H*, a SCAD-based penalty for similarity control, as well as a jackknife-based standard error estimation. This framework enjoys several advantages over existing methods. First of all, compared to scRNA-seq, SCADIE’s cell type proportion estimate is not affected by technical dropouts. Second, SCADIE has shown better sensitivity and specificity compared to existing methods. In addition, SCADIE also has certain advantages in functionality compared to other methods mentioned in this paper: when compared to CIBERSORTx, SCADIE’s design can estimate and test all the genes to identify DEGs (while CIBERSORTx only imputes a subset of given genes); it can also accommodate more than microarray data and output matched cell proportions (while csSAM only works on microarray data); when compared to TOAST, SCADIE can not only perform hypothesis testing for DEGs, but produce matched point estimates in the meantime. It is worth mentioning that recently two additional methods, CellR [5] and CARseq [12], have been proposed to specifically perform cell type-specific DEG identification based on RNA-Seq raw counts data. We were unable to include them in our benchmarking because most of the data in our study were only available either as normalized RNA-Seq or microarray, but they might have superior performance in heavily normalized brain tissue data where SCADIE’s performance was not ideal.

Through extensive comparisons using simulated and real datasets, we demonstrated that SCADIE can not only maintain high cell proportion estimate accuracy, it can also effectively identify cell type-specific DEGs. In the cases where initialization quality is poor or with barely sufficient sample size, SCADIE’s performance will be affected; however, its iteration procedure can still recover signals to certain extent. We have provided guidelines regarding SCADIE’s applicability under these extreme situations. Besides, its performance is highly robust with respect to a range of parameter values. These features all ensure that SCADIE can be broadly applicable to different settings.

Although SCADIE enjoys several advantages over existing methods, there are several aspects that can be further explored. First, the proposed NMF estimate may involve bias due to the non-negative constraint and the penalization term. One can consider a de-biased estimate to fix the potential bias of the proposed penalization method to improve inference. Second, although SCADIE can take initial input from any deconvolution algorithm, it only uses NNLS in its iterative *H*-update step. We may expand its modularity and enable it to fully take advantage of other deconvolution methods (e.g., DWLS for cases with rare cell types, and weighted NNLS [42] for single cell counts data). It shall also be noted that although SCADIE is built as a supervised deconvolution tool, it is also compatible with all the unsupervised deconvolution methods that only require bulk gene expression data [13, 31, 44] as long as they could provide initial *W* and *H*. Unsupervised methods are useful in situations of cell type discovery or lack of supervising information, but as there is no guarantee that their inferred cell types have one-to-one mapping to actual cell types, annotating cell types remains a challenge. Third, we have shown that using the full *W* along with NNLS has provided robust and accurate *H* estimates over iteration, further investigation into the mechanism behind this may enlighten the simplification of deconvolution. Finally, we can improve the DE hypothesis testing procedure by incorporating more advanced DE techniques and better false discovery control into the framework.

## Conclusions

Simultaneous estimation of cell type proportions and cell type-specific gene expressions from bulk gene expression data remains a challenge due to its non-identifiable nature. In this article, through our proposed method SCADIE, we demonstrated that with reasonable assumptions on the similarity between group level cell type-specific gene expression profiles, proper design of objective function, and reasonable initial deconvolution accuracy, it is possible to infer cell type proportions along with cell type-specific gene expressions with robustness and high accuracy. Despite this progress, technical challenges including multi-group comparison, limited sample size, and poor initialization quality still remain to be further addressed in the future.

## Methods

For a full list of notations, refer to Additional File 1: Supplementary section S1.

### Rationale behind SCAD penalty

A key consideration in our proposed method is to maintain a proper dissimilarity level between *W*_1_ and *W*_2_, where the true DEGs can be identified without introducing many false-positive DEGs. In our simulation analyses, we found that keeping *W*_1_ and *W*_2_ similar by a ridge penalty did increase the accuracy in *H*s (Additional File 1: Supplementary Fig. S1cd). However, the *W* accuracy and DEG identifying power are reduced (Additional File 1: Supplementary Figs. S1ab, S2). An intuitive explanation to this is that forcing *W*_1_ and *W*_2_ prevents them from being too divergent with each other, thus increasing *H* accuracy; however, this penalty also makes *W*_1_ and *W*_2_ over-similar, thus reducing its sensitivity substantially (Fig. S2).

To combine the advantages with and without ridge penalty, we adopt the SCAD-based penalty that imposes entry-specific dissimilarity penalty based on the prior difference between *W*_1_ and *W*_2_: if the separately estimated $\bar {W}_{1}$ and $\bar {W}_{2}$ have similar entries for the (*k*,*j*) component, we put a high but bounded penalty on their difference in our procedure, whereas if the components are quite different, we penalize less on the difference. Specifically, we penalize $\sum _{k,j} E_{jk} \left ([W_{1}]^{T}_{jk} - [W_{2}]^{T}_{jk}\right)^{2}$, where $E_{jk} = P'_{\zeta _{n}}\left \{ \left [\bar {W}_{1}^{T} - \bar {W}^{T}_{2}\right ]^{2}_{jk}\right \}$ and $P^{\prime }_{\zeta _{n}}(\cdot)$ is the derivative of the SCAD penalty function as defined in (6). To the best of our knowledge, weighted *ℓ*_2_ penalty using the derivative of SCAD has not been investigated. By doing so, we can incorporate the structure pattern in $\bar {W}_{1} - \bar {W}_{2}$ when estimating *W*_1_ and *W*_2_ in a more adaptive manner (see the sections “Warm-up run and weight matrix derivation” and “Update *W* and *H*” for more details).

Theoretical analyses suggest that this novel penalty structure can achieve high accuracy in the estimation of *W* and *H* (Additional File 1: Supplementary section S3.1). In addition, we performed simulations comparing sensitivity, specificity, and positive predictive rate (PPV) by using the following: (1) independent *W*_1_,*W*_2_ updates with NNLS, (2) ridge regression imposing similarity between *W*_1_ and *W*_2_, and (3) SCAD-penalty. The results suggest that SCAD-penalty can keep sensitivity high while in the meantime better control false positives through its precise penalty (Figs. S1 and S2).

### Initialization

For SCADIE’s initialization, users can either input bulk matrices along with corresponding *H*s, or input bulk and signature matrices to perform generic deconvolution using NNLS or DWLS[40]. Given that many deconvolution methods have been proposed to accommodate various data and conditions, we recommend users provide bulk matrix along with the best initial *H*s available.

In real applications, obtaining accurate deconvolution results is often difficult. We have shown that although poor initialization does affect SCADIE’s performance, its iterative procedure could recover the signals and produce decent results (see the section “SCADIE improves AD-associated cell type-specific DEG estimation” and Fig. 8).

### Warm-up run and weight matrix derivation

The proposed penalty requires a prior weight matrix *E* of the same dimension as *W*, which provides prior information on how likely certain entries differ between two *W*s.

To obtain *E*, we perform a few steps of “warm-up” iterations:

1. We first obtain full $W_{1}^{(0)}$ and $W_{2}^{(0)}$ from solving the NNLS problem $\min _{W_{i} \in \mathbb {R}_{+}^{m \times k}} \left \|Y_{i}^{T} - H_{i}^{T}W_{i}^{T} \right \|_{F}^{2}, i = 1,2$.
2. The *H*s are subsequently updated by NNLS using full *Y*s and *W*s.
3. Repeat steps 1–2 for a few rounds (default 5 rounds, but can be manually changed), and plug in the output $\hat {W}_{1}^{NNLS},\hat {W}_{2}^{NNLS}$ to Eq. 6 in the section “Update *W* and *H*” to obtain the weight matrix *E*.

### Update *W* and *H*

In this subsection, we provide details of updating *W* and *H*, which corresponds to step (b) in Fig. 1.

Note that for the simplicity of notation, we intrinsically assume the sample sizes of group 1 and group 2 are both *n* throughout analyses in this paper, i.e., *Y*_1_,*Y*_2_∈*R*^(*m*×*n*)+^ and *H*_1_,*H*_2_∈*R*^(*k*×*n*)+^. Although this is not the case in most real applications, making this assumption does not affect either our theoretic derivation or most implementations. For scenarios where *n*_1_≠*n*_2_ makes a difference (e.g., jackknife estimation), we will discuss our handling of this issue specifically.

For the update of *H* in the main iterative procedure, we simply use NNLS to solve for the problems below for the two groups separately:

4 $$\begin{array}{*{20}l} \min_{H_{i} \in \mathbb{R}^{(k \times n)+}} \|Y_{i} - W_{i} H_{i} \|_{F}^{2}, i = 1,2. \end{array} $$

Note that here we use the full *W*s instead of signature genes only, and this alteration still produces good estimation accuracy (see the section “SCADIE maintains high cell proportion estimation accuracy”).

To update *W*_1_ and *W*_2_ simultaneously, we consider the following weighted-regression-based optimization:

5 $$  \min_{W_{1}, W_{2} \in \mathbb{R}^{(m \times k)+}} \frac{1}{2} \left\|Y_{1}^{T} - H^{T}_{1} W_{1}^{T}\right\|_{F}^{2} + \frac{1}{2} \left\|Y_{2}^{T} - H^{T}_{2} W_{2}^{T}\right\|_{F}^{2} + \frac{\lambda}{2} \left\|W_{1}^{T} - W_{2}^{T}\right\|_{E,F}^{2},  $$

where $\|W_{1}^{T}-W_{2}^{T}\|_{E,F}^{2} = \sum _{j,k} E_{jk} \left (W_{1}^{T}-W_{2}^{T} \right)_{jk}^{2}$. Note that (5) is not separable with respect to *W*_1_ and *W*_2_ due to the third term, from which the information is shared between the two groups.

Let $\tilde {W} = [{W}_{1}, {W}_{2}]^{T} \in \mathbb {R}^{(2k \times m)+}$. We update each column of $\tilde {W}$ separately. Specifically, updating the *j*th column of $\tilde {W}$ is equivalent to solving

$$\hat{x}^{(j)} = \arg\min_{x \in \mathbb{R}_{+}^{2k \times 1}} \left\|\tilde{Y}^{(j)} - \tilde{X}^{(j)} x\right\|_{F}^{2}, $$

where

$$\tilde{Y}^{(j)} = \left[\begin{array}{c} [Y_{1}^{T}]_{j} \\ \left[Y_{2}^{T}\right]_{j} \\ {0}_{k,1}\\ \end{array}\right] \in \mathbb{R}^{(2n+k) \times 1}, \quad \tilde{X}^{(j)} = \left[\begin{array}{cc} H_{1}^{T} & 0_{n,k} \\ 0_{n,k} & H_{2}^{T}\\ \sqrt{\lambda} \text{diag}(\sqrt{E_{j}}) & - \sqrt{\lambda} \text{diag}(\sqrt{E_{j}})\\ \end{array}\right], $$

where *A*_*j*_ represents the *j*th column of a matrix *A*. Then the minimizer $\hat {x}^{(j)}$ corresponds to the *j*th column of $\tilde {W}$.

For the weight matrix $E \in \mathbb {R}^{k \times m}$, we set $E_{jk} = P'_{\zeta _{n}}\left \{ \left [\bar {W}^{T}_{1} - \bar {W}_{2}^{T}\right ]^{2}_{jk}\right \}$, where $P^{\prime }_{\zeta _{n}}(\cdot)$ is the derivative of the SCAD penalty function, i.e.,

6 $$\begin{array}{*{20}l}  P'_{\zeta_{n}}(x) = I(x \le \zeta_{n}) + \frac{(a\zeta_{n} - x)_{+}}{(a-1)\zeta_{n}} I(x > \zeta_{n}), \end{array} $$

with a regularization parameter *ζ*_*n*_≥0, and $\bar {W}_{1}$ and $\bar {W}_{2}$ are the separate estimates obtained from the previous step. We set *a*=3.7 as suggested by [8], which is known to be optimal based on cross-validated empirical studies [20]. For the choice of parameter *ζ*_*n*_, we keep *ζ*_*n*_=4 throughout all our analyses. We also demonstrate that SCADIE output is robust with respect to *ζ*_*n*_ in terms of *H*s, *W*s, and DEG identification, if *ζ*_*n*_ is in an appropriate range. See the section “Robustness with respect to *ζ*_*n*_” and Additional File 1: Supplementary section S3.2 for more details.

The proposed weighted-regression-based optimization (5) with the weight based on SCAD derivative function can be understood as the one-step local linear approximation of the following SCAD penalty [45]:

$${}\min_{W_{1}, W_{2} \in \mathbb{R}^{(m \times k)+}} \frac{1}{2} \left\|Y_{1}^{T} - H^{T}_{1} W_{1}^{T}\right\|_{F}^{2} + \frac{1}{2} \left\|Y_{2}^{T} - H^{T}_{2} W_{2}^{T}\right\|_{F}^{2} + \sum_{j,k} P_{\zeta_{n}} \left(\left[W_{1}^{T} - W_{2}^{T}\right]^{2}_{jk}\right). $$

Hence, the proposed weighted regression-based optimization penalizes differently based on the range of $[W_{1}^{T} - W_{2}^{T}]_{jk}$. Compared with the SCAD penalty, the proposed method can be efficiently computed and enjoys the same theoretical properties known as “oracle properties” [45].

The SCAD penalty and weighted *ℓ*_1_ penalty using the derivative of SCAD are widely used in the penalization methods [7, 21]. SCAD enjoys variable selection consistency and unbiasedness property by imposing no weights on the signal that is beyond 3.7*ζ*_*n*_, which may reduce bias. By using the weighted Frobenius penalization, we may obtain less biased estimate compared to that of Frobenius penalization. In theory, we derive the estimation error bound of the proposed method under certain regularity conditions if *ζ*_*n*_ is in an appropriate range; see Additional File 1: Supplementary section S3.1 for more details.

### Jackknife standard error estimation

The above iterative procedure only provides us with point estimates of *W*_1_,*W*_2_,*H*_1_, and *H*_2_. For differential expression analysis between *W*_1_ and *W*_2_, we use a leave-one-out jackknife procedure to estimate standard error for *W*_1_−*W*_2_.

For standard error estimation in most real data, *Y*_1_ and *Y*_2_ have different sample sizes (number of columns). We denote *n*_1_ and *n*_2_ as column numbers of *Y*_1_,*Y*_2_, respectively, and let *n*_0_= min{*n*_1_,*n*_2_}. Then we run the iterative procedure *n*_0_ times—each time leaving one sample out from *Y*_1_,*H*_1_ and *Y*_2_,*H*_2_, respectively; this will give us *n*_0_ different $\hat {W}_{1}-\hat {W}_{2}$s, and element-wise jackknife standard error estimates can be obtained by: $\sigma ^{jackknife}_{ij} = \frac {n_{0}-1}{\sqrt {n_{0} }}\hat {\sigma }_{ij}, i=1,...,m; j = 1,...,k$, where $\hat {\sigma }_{ij}$ is the sample standard deviation for the *ij*th entry from the $n_{0} \hat {W}_{1}-\hat {W}_{2}$s [23]. With these, we can then conduct element-wise hypothesis testing to identify DEGs between *W*_1_ and *W*_2_.

We compared the jackknife standard error estimates with those from bootstrap, and both led to highly consistent results (Additional File 1: Supplementary section S3.6). In the R package implementation of SCADIE, bootstrap is also available for standard error estimation. However, we recommend using jackknife for general purposes to avoid the potential singularity issue arising from bootstrap’s sampling with replacement; see Additional File 1: Supplementary section S3.6 for a detailed explanation.

### Simulation models and benchmarking

#### Simulation datasets

The simulation data used in the section “SCADIE maintains high cell proportion estimation accuracy” to section “SCADIE can improve the estimates from other methods” were generated as follows: first, *W*_1_∈*R*^5000×5^ was generated with all its entries following the log-normal distribution with mean 8 and standard deviation 3; then to generate *W*_2_, 2.5% of the entries in *W*_1_ were upregulated to 1.5× or 2×, with another 2.5% downregulated to 0.67× or 0.5×; *H*_1_ and *H*_2_ were generated using two distinct Dirichlet distributions, each group consisting of 20 samples; bulk expression matrices *Y* were generated by *W*·*H*+*ε*, where *ε* is a Gaussian white noise matrix with *sd*=4, if negative entries were present after adding noise, these entries were reset back to 0.

For signature matrix generation, we first obtained $\bar {W}=\frac {W_{1}+W_{2}}{2}$ and used the top 5% rows in terms of the largest-entry/second-largest entry ratio as signature gene rows. In *H*’s benchmarking, all methods support *H* estimation with bulk gene expression and signature matrix as input; since MuSiC only supports count data, we rounded the data before input into MuSiC.

#### Mouse ISC pseudo-bulk data set

The pseudo-bulk data used in the section “SCADIE maintains high cell proportion estimation accuracy” to section “SCADIE can improve the estimates from other methods” were generated as follows. We downloaded the original ISC scRNA-seq data through GEO using accession number GSE92865. We clustered its 14 cell types into four major cell types: ISC, TA, Ent, and other, which was based on the t-SNE results of the paper [43]. We then separated the scRNA-seq data by treatment status, where the Fc treatment was group 1, the scFv-DKK1c treatment was group 2, and the RSPO2 treatment was group 3. The corresponding *W*_1_,*W*_2_,*W*_3_ matrices were generated by averaging over all cells in each major cell type of the same treatment group. *H*_1_ to *H*_3_ were generated the same way as previous section, also with 20 samples in each group. Finally, *Y*_1_ to *Y*_3_ were generated by *Y*_*i*_=*W*_*i*_·*H*_*i*_+*ε*,*i*=1,2,3, where *ε* is a Gaussian white noise matrix with *sd*=4.

Signature matrix was derived using the *buildSignatureMatrixUsingSeurat* function from the DWLS package [32] using all the single cells regardless of their treatment status.

Ground truth DEGs were derived by performing differential expression analysis for each cell type between group 2 and group 1 and group 3 and group 1, using the *DEAnalysis* function in the DWLS package [40].

In the sections “SCADIE can better identify DEGs” and “SCADIE can improve the estimates from other methods,” mouse ISC pseudo-bulk 1 dataset consists of group 1 and group 2 data, while the pseudo-bulk 2 dataset consists of group1 and group3 data.

#### Mouse bulk data set

In thesection “SCADIE maintains high cell proportion estimation accuracy,” we used a mouse brain-liver-lung mixture microarray dataset (referred as mouse bulk); the data were accessed through GEO using accession number GSE19830. Raw data were preprocessed with the *affy* package in R and normalized using the rma method. We used rma normalization to keep the data comparable to [34]. The signature matrix was generated using the DWLS package [40]. In *H*’s benchmarking, since MuSiC only supports count level data, we rounded the bulk matrices and signature gene matrices before input into MuSiC.

### Real data processing and analyses

#### COPD single cell and bulk data

Raw scRNA-seq data were obtained from [1]. Data preprocessing, quality control, and normalization were done using Seurat V3 [36] in R. The original data contained samples of control, COPD, and idiopathic pulmonary fibrosis (IPF), where cells from IPF samples were excluded in our analysis. There were 37 distinct cell types originally; we used the five major cell type clusters according to UMAP clustering from [32], and we did not further sub-divide because (1) there are limited cell numbers in some clusters and (2) the correlated expression profiles of cell types within each cluster might introduce unwanted collinearity in *W*. “Groundtruth” DEGs were identified for each cell type between control and COPD using the DWLS package[32].

Signature matrices were generated using the CIBERSORTx [25] and DWLS packages [40]. The cell type proportion results from DWLS were considered better, and we proceeded with its signature matrix.

Bulk RNA-Seq data in FPKM was obtained through GEO with accession code GSE57148, the FPKM matrix was further transformed into log scale to accommodate the scale of signature matrix.

For benchmarking with TOAST, we input the bulk gene expression and the same initial *H*s as SCADIE; the control and COPD groups were denoted as groups 0 and 1, respectively. We used the output effect size as indication of DEGs’ direction, and *p*-value to identify significant DEGs.

For GSEA analysis, we first transformed the single cell and SCADIE-derived DEGs into *z*-scores. For single cell DEGs, the *z*-scores were obtained from normal distribution quantiles of their *p*-values, while for SCADIE output, since we know the point estimates and standard error estimates for all genes, we directly calculated their *z*-scores. The *z*-score lists for both groups were sorted and input for pre-ranked GSEA analysis using R’s *fgsea* package, and Molecular Signatures Database v7.4 database [17]. For the top enrichment analysis, pathways of each group were first ranked by their GSEA *p* values, then the top 5% or 10% were chosen and compared accordingly. The overlapping *p*-values were calculated using a binomial distribution, where the model parameter *N* equals the total number of shared pathways, while *p*=0.01 for top 10% overlapping and *p*=0.0025 for top 5% overlapping under the null hypothesis.

#### DLBLC data

Raw bulk microarray data were obtained through GEO with accession number GSE10846. Raw data were preprocessed with *affy* package in R and normalized using mas5 method. Sample treatment and subtype information was retrieved using the *GEOquery* package. LM22 matrix from [25] was used for initial deconvolution.

#### Alzheimer’s disease data

Bulk post-mortem RNA-Seq samples of prefrontal cortex were downloaded from the ROSMAP cohort [3]. We only kept the subset of 49 samples whose cell type proportion results were measured in [27]. The IHC results measured four major cell types (neuron, astrocyte, oligodendrocyte and microglia) without differentiating between excitatory and inhibitory neurons, while the cell type-specific DEGs from [22] did separate these two cell types. In this regard, we only included results for the three overlapping cell types.

#### Follicular lymphoma data

The raw bulk microarray data were accessed through GEO with accession number GSE127462; preprocessing and normalizing were performed the same as the above procedure for DLBLC. The initial deconvolution used LM22 matrix from [25] and we merged the cell types into the following 8 major groups (B cell, CD8 T CELL, CD4 T cell, NK, Monocyte/Macrophage, DC cell, Mast, Neutrophil) before inputting this updated *H* into SCADIE. For the DEGs comparison, unlike SCADIE’s whole transcriptome approach, we ran CIBERSORTx’s high resolution mode only inputting those known DEGs. For TOAST run, we input bulk gene expression and initial the same initial *H*s as SCADIE and CIBERSORTx; WT group was denoted as group 0, and MT group was denoted as group 1. The output DEGs’ directions were determined by their effect sizes; *p*-values were directly from output. Noted that in our analysis for COPD, we kept its original effect sizes as fold change measure, instead of trying to transform to log2 fold change.

## Supplementary Information

**Additional file 1** Includes notations, additional figures, theoretical properties of the proposed method, simulation models, details of the simulation and real data processing.

**Additional file 2** Supplementary Table S1 contains details on the DEGs exclusively identified by SCADIE as discussed in Section ‘SCADIE accurately infers cell proportions and cell type-specific genes in Chronic Obstructive Pulmonary Disease’.

**Additional file 3** Review history.

## Data Availability

The code for SCADIE is available at https://github.com/tdw1221/SCADIE under MIT license and the source code used in this paper is available at Zenodo [38] under Creative Commons Attribution 4.0 International license. The original scRNA-seq data used for generating pseudo-bulk data used in the section “SCADIE maintains high cell proportion estimation accuracy” to section “SCADIE can improve the estimates from other methods” can be accessed through GEO with accession number GSE92865. The original microarray data used in the section “SCADIE maintains high cell proportion estimation accuracy” can be accessed through GEO with accession number GSE19830. For COPD analyses, raw scRNA-seq data can be obtained from [1], and bulk RNA-Seq data in FPKM can be obtained through GEO with accession number GSE57148. DLBLC data can be accessed through GEO with accession number GSE10846. For analyses performed in the section “SCADIE improves AD-associated cell type-specific DEG estimation,” bulk post-mortem RNA-Seq samples can be downloaded from the ROSMAP cohort [3]. The raw bulk microarray data of follicular lymphoma in the section “DEG identification under poor initialization and limited sample size” can be accessed through GEO with accession number GSE12746.
