# Supplementary material for: SCADIE: simultaneous estimation of cell type proportions and cell type-specific gene expressions using SCAD-based iterative estimating procedure
Source: Genome Biol. 2022 Jun 15;23:129. doi: 10.1186/s13059-022-02688-w (PMC9199219; doi:10.1186/s13059-022-02688-w)
Supplement: Supplementary file 1 — Additional file 1 Includes notations, additional figures, theoretical properties of the proposed method, simulation models, details of the simulation and real data processing. [file 13059_2022_2688_MOESM1_ESM.pdf]

Additional File 1: SCADIE: simultaneous estimation of cell type proportions and cell type-specific gene expressions using SCAD-based iterative estimating procedure

Daiwei Tang, Seyoung Park, and Hongyu Zhao

May 6, 2022

Supplementary files include notations, additional figures, theoretical properties of the proposed method, and sensitivity analysis.

S1 Notations

$Y \in R^{(m\times n)+}$: bulk gene expression matrix, each row represents a gene and each column represents a sample. When there are two groups of samples with different sample sizes, we denote $Y_1 \in R^{(m\times n_1)+}$ and $Y \in R^{(m\times n_2)+}$.
$W \in R^{(m\times k)+}$: cell type-specific gene expression profile, each row represents a gene and each column represents a cell type.
$H \in R^{(k\times n)+}$: cell types proportion matrix, each row corresponds to a given cell type's proportion and each column is a sample. When there are two groups of samples with different sample sizes, we denote $H_1 \in R^{(k\times n_1)+}$ and $H_2 \in R^{(k\times n_2)+}$.
$\underline{Y}$: sub-Y matrix with only rows containing signature genes.
$\underline{W}$: sub-W matrix with only rows containing signature genes.
$\tilde{\tilde{W}}$: weighted-W matrix where each row is given different weight based on their cell type differentiating power.
$\tilde{Y}$: transpose of bulk gene expression matrix Y.
E: weight matrix used for SCAD-penalty, E is of the same dimension as W^T.
$\bar{W}_k$: separate estimate of W using the k-th group for $k = 1, 2$.
W_k^o, H_k^o: the underlying profile and the proportion matrix in the theoretical model $Y_k = W_k^0 H_k^0 + \varepsilon_k$ for $k = 1, 2$.
$\hat{W}_k$: the proposed estimate of W_k^o for $k = 1, 2$.
$\tilde{W} = [W_1, W_2]^T \in \mathbb{R}^{(2k\times m)+}$.
$\Sigma_{W_1-W_2}$: the entry-specific standard deviation matrix for $W_1 - W_2$, when referring to its element, we used $\sigma_{W_1-W_2}$.

S2 Supplementary Figures and Tables

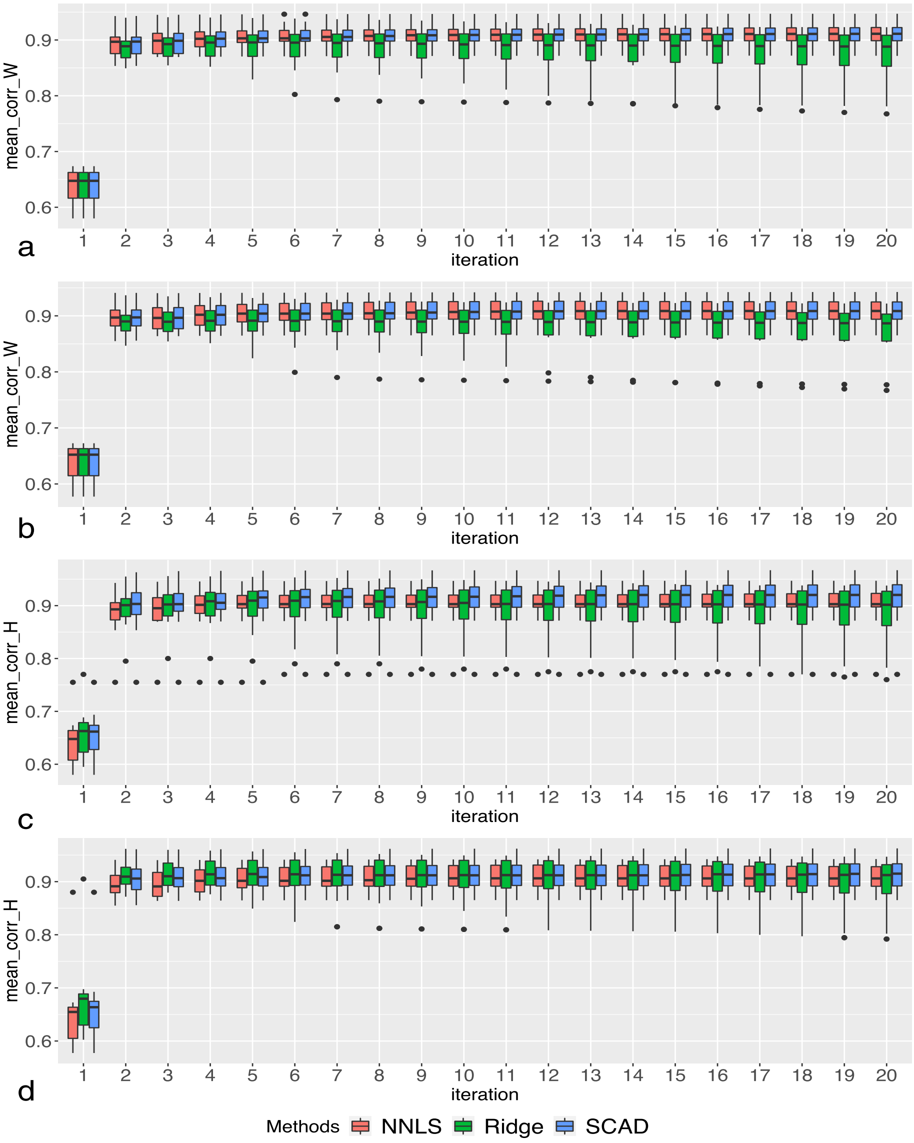

Figure S1: W and H accuracy over iteration for three W-update methods: NNLS, ridge regression, and SCAD-penalized regression. a. W_1 accuracy over iteration; b. W_2 accuracy over iteration; c. H_1 accuracy over iteration; d. H_2 accuracy over iteration.

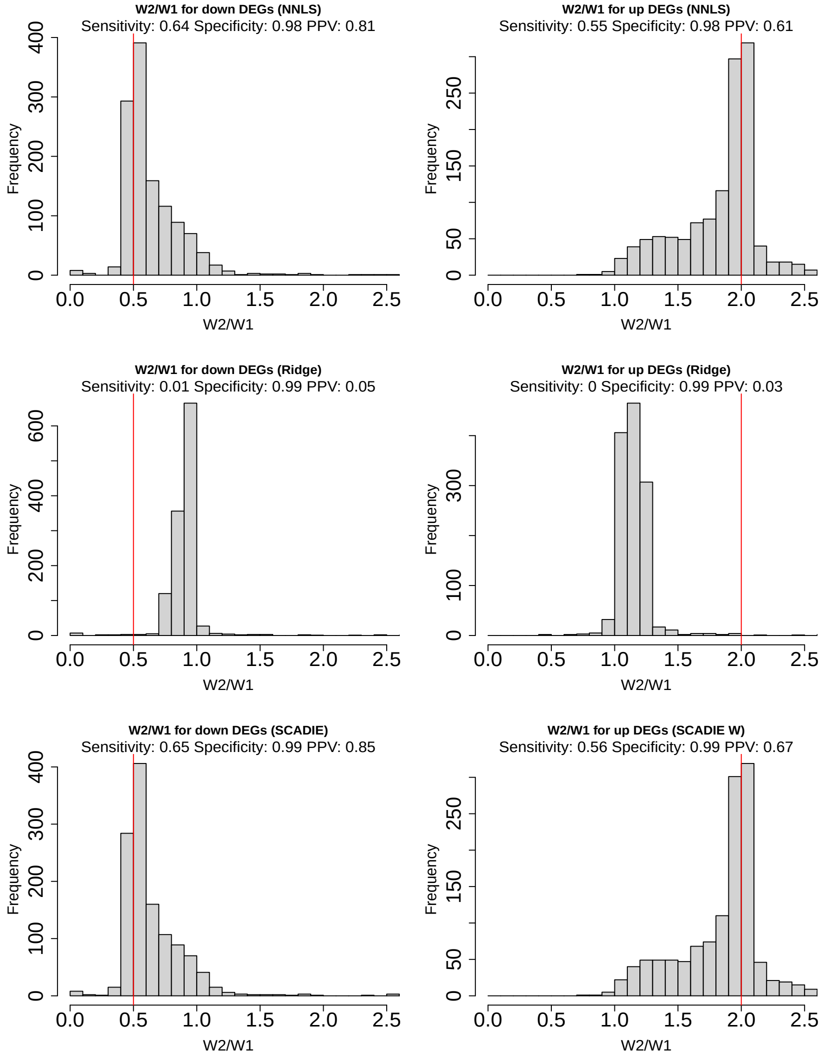

Figure S2: Histograms showing the estimated W_2/W_1 for known up-regulated and down-regulated DEG entries from three W-update methods: NNLS, ridge regression and SCAD-penalized regression. Sensitivity, specificity and positive predictive rate (PPV) are shown under each figure's title. Red vertical lines indicate the true fold change level. Due to the matrix-wise dissimilarity penalty in ridge regression, sensitivity from ridge regression is extremely low. Both independent W-update through NNLS and SCAD-penalty achieve high sensitivity, but SCAD has better false positive control (shown by higher PPV) due to its entry-specific precise penalty, this is also lined up with results from Figure 2. As the vast majority of entries are non-DEGs, specificity in this case is less meaningful than PPV.

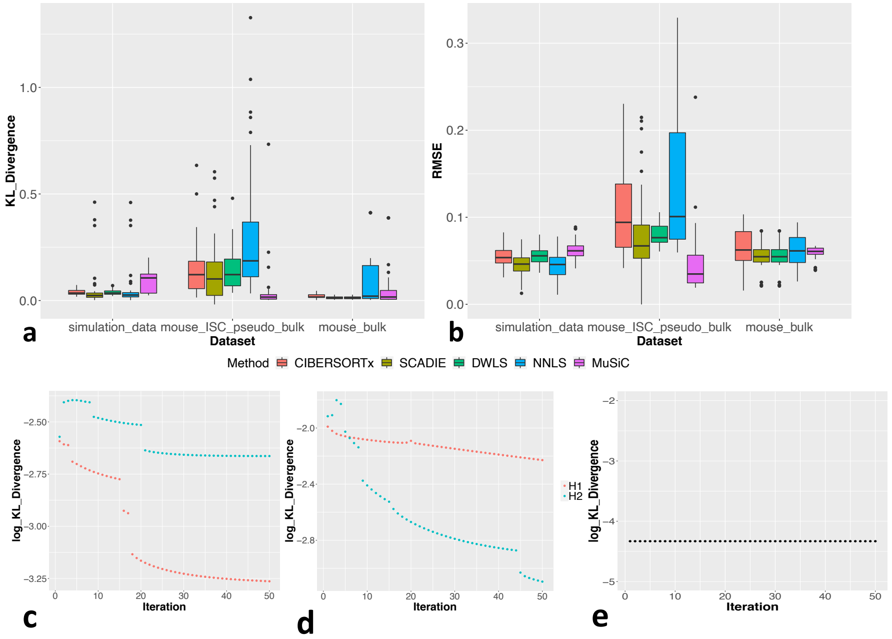

Figure S3: Benchmark cell type proportion estimations from SCADIE against DWLS, CIBERSORTx, MuSiC, and the naive iterative procedure with NNLS W-update: a. K-L Divergence between H and the ground truth proportions across three data sets, SCADIE and NNLS iteration's Hs were from the final iteration output, and Hs of DWLS and CIBERSORTx were directly from deconvolution; b. Same results as a) but measured by root-mean-square error (RMSE), the result patterns are consistent with those in K-L Divergence; c-e: H accuracy over iterations, where figure b is simulation dataset, figure c is pseudo-bulk dataset, figure d is bulk microarray dataset.

Cell	#DEGs from single cell (SC)	%DEGs Correct Direction from SCADIE	%Correctly Identified SC DEGs	#Additional DEGs from SCADIE
Stromal	437	61.6	31.1	1106
Myeloid	216	77.3	32.9	1114
Lymphoid	75	64.0	21.3	542
Epithelial	428	67.8	9.3	41
Endothelial	293	75.8	27.3	69

Figure S4: A summary table comparing single cell-derived DEGs and SCADIE-inferred DEGs between COPD and control samples, the column " %DEGs Correct Direction from SCADIE " represents the percentages of single cell DEGs that were of concordant directional changes from bulk data inferred by SCADIE, the column "%SC Correctly Significant DEGs identified from SCADIE" represents the percentages of single cell DEGs that were also identified as significant DEGs from bulk data by SCADIE, the column " #Additional DEGs from SCADIE" represents the DEGs SCADIE identified from bulk data that were not identified by single cell DEG.

	Stromal	Myeloid	Lymphoid	Epithelial	Endothelial
#MSigDB pathways present in both outcomes	21507	18670	12447	20744	18826
Expected overlapping (Top 5%)	53	47	31	52	47
Actual overlapping (p-value)	149 ($p < 10^{-5}$)	128 ($p < 10^{-5}$)	46 ($p = 5 \times 10^{-3}$)	179 ($p < 10^{-5}$)	58 ($p = 0.05$)
Expected overlapping (Top 10%)	215	187	124	207	188
Actual overlapping (p-value)	422 ($p < 10^{-5}$)	332 ($p < 10^{-5}$)	205 ($p < 10^{-5}$)	479 ($p < 10^{-5}$)	248 ($p = 1.2 \times 10^{-5}$)

Figure S5: A summary table comparing GSEA outcomes for single cell and SCADIE derived DEG lists. The first row indicates the number of MSigDB pathways that present in both single cell and SCADIE GSEA outcomes. The 2nd-3rd rows indicate the expected number of overlapped pathways under null hypothesis, and the actual overlappings along with p-values for the top 5% pathways. The 4th-5th rows indicate the expected number of overlapped pathways under null hypothesis, and the actual overlappings along with p-values for the top 10% pathways.

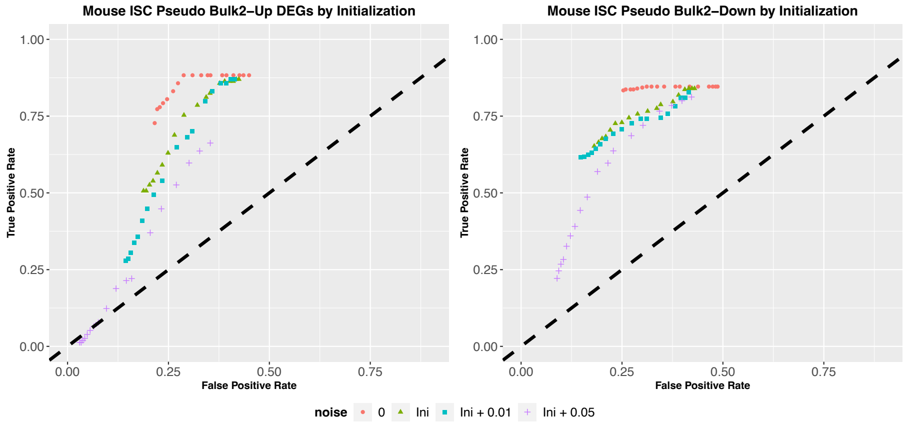

Figure S6: Sensitivities and false positive rates for up-regulated and down-regulated DEGs over a range p values under initialization: "0" is using the ground truth H for initialization, "Ini" is the same SCADIE initialization used in Section "SCADIE can better identify DEGs", "Ini + 0.01" refers to additional sd=0.01 Gaussian noise was added to Ini's H and each column of H was re-balanced to 1, similar for "Ini + 0.05" where the sd = 0.05.

S3 Supplementary Results

S3.1 Theoretical properties

Because NNLS is used when updating W and H, in this subsection, we develop a general theory for the NNLS estimate under some regularity conditions. Let $\hat{w}$ and $\tilde{w}$ be the NNLS and Ordinary Least Squares (OLS) estimators, respectively. Even in the non-negative regression coefficient setting, we can empirically check that there is no guarantee that the error $\|\hat{w} - w^o\|$ is less than $\|\tilde{w} - w^o\|$, where w^o is the underlying non-negative coefficient vector. For example, we considered a linear regression model $y = Xw^o + \epsilon$, where $m = 300$, $k = 5$, entries in X's are i.i.d. N(0,1), and $[w^o]_j$'s are i.i.d. Uniform(0,5). We observed that $\|\hat{w} - w^o\|$ is greater than $\|\tilde{w} - w^o\|$ for about 30% of the cases. With this regard, the bound in Theorem 1 is not trivial.

Theorem 1. *[General estimation error bound] Suppose that $y = Xw^o + \epsilon$, where $y \in \mathbb{R}^m$ is a response vector, $X \in \mathbb{R}^{m \times k}$ is observable and its columns have full-rank, and $w^o \in \mathbb{R}^k_+$, i.e., its components are non-negative. Suppose that $\kappa(X^T X) := \lambda_{\max}(X^T X)/\lambda_{\min}(X^T X) \leqslant M$ for some constant $M > 0$. Let $\hat{w}$ be the non-negative least squares estimate, i.e.,*

$$\hat{w} = argmin_{w \in \mathbb{R}^k_+} \|y - Xw\|^2.$$

Then, the estimation error of $\hat{w}$ satisfies $\|\hat{w} - w^o\| \leqslant \sqrt{M}\|\epsilon\|$.

Proof. Recall OLS estimate $\tilde{w} = (X^T X)^{-1} X^T y$. Then, for any $w \in \mathbb{R}^k$, it holds that

$$\|y - Xw\|^2 = \|y - X\tilde{w}\|^2 + \|X(w - \tilde{w})\|^2.$$

Thus, we have

$$\hat{w} = \text{argmin}_{w \in \mathbb{R}_+^k} \|X(w - \tilde{w})\|^2.$$

Note that $K = \{Xw : \ w \in \mathbb{R}_+^k\}$ is a closed convex cone. Then, for a projection mapping $p(\cdot)$ onto K, we can write

$$X\hat{w} = p(X\tilde{w}), \quad Xw^o = p(Xw^o).$$

Since $p(\cdot)$ is non-expansive as in Lemma 3 of [11], we have

$$\|X(\hat{w} - w^o)\| = \|p(X\tilde{w}) - p(Xw^o)\| \leqslant \|X(\tilde{w} - w^o)\|.$$

Thus

$$\begin{aligned} \lambda_{\min}(X^T X)\|\hat{w} - w^o\|^2 &\leqslant (\hat{w} - w^o)^T X^T X(\hat{w} - w^o) \leqslant (\tilde{w} - w^o)^T X^T X(\tilde{w} - w^o) \\ &\leqslant \lambda_{\max}(X^T X)\|\tilde{w} - w^o\|^2. \end{aligned}$$

Hence,

$$\|\hat{w}-w^o\| \leqslant \sqrt{\kappa(X^T X)}\|\tilde{w}-w^o\| \leqslant \sqrt{M}\|\tilde{w}-w^o\| = \sqrt{M}\|X(X^T X)^{-1}X^T\epsilon\| \leqslant \sqrt{M}\|\epsilon\|,$$

where the last inequality follows from the fact that $X(X^T X)^{-1}X^T$ is a projection matrix. This completes the proof. □

The following Corollary 1 shows theoretical properties of the proposed method.

Corollary 1. *[Estimation error bound of $\hat{W}_i$ for $i = 1, 2$] Suppose that $Y_1 = W_1^0 H_1^0 + \varepsilon_1$ and $Y_2 = W_2^0 H_2^0 + \varepsilon_2$. Let $\Omega := \{(j,k) : \ [W_1^0]_{jk}^T \neq [W_2^0]_{jk}^T\}$. Suppose that the separate estimates $\bar{W}_1$ and $\bar{W}_2$ satisfy $3.7\zeta_n \leqslant \min_{(j,k)\in\Omega} |[\bar{W}_1]_{jk}^T - [\bar{W}_2]_{jk}^T|$. Then, the proposed estimates $\hat{W}_1$ and $\hat{W}_2$ satisfy*

$$\|\hat{W}_1 - W_1^0\|^2 + \|\hat{W}_2 - W_2^0\|^2 \leqslant M(\|\varepsilon_1\|^2 + \|\varepsilon_2\|^2),$$

where $M = \kappa\left[\begin{pmatrix} H_1 H_1^T + \lambda diag(E_j) & -\lambda diag(E_j) \\ -\lambda diag(E_j) & H_2 H_2^T + \lambda diag(E_j) \end{pmatrix}\right].$

Proof. Recall that updating the jth column of $\tilde{W} = [W_1, W_2]^T$ is equivalent to solving

$$\hat{x}^{(j)} = \arg\min_{x \in \mathbb{R}_+^{2k\times 1}} \|\tilde{Y}^{(j)} - \tilde{X}^{(j)}x\|_F^2,$$

where

$$\tilde{Y}^{(j)} = \begin{bmatrix} [Y_1^T]_j \\ [Y_2^T]_j \\ 0_{k,1} \end{bmatrix} \in \mathbb{R}^{(2n+k)\times 1}, \quad \tilde{X}^{(j)} = \begin{bmatrix} H_1^T & 0_{n,k} \\ 0_{n,k} & H_2^T \\ \sqrt{\lambda}\text{diag}(\sqrt{E_j}) & -\sqrt{\lambda}\text{diag}(\sqrt{E_j}) \end{bmatrix}.$$

Thus, by Theorem 1, for each $j = 1, \cdots, m$, $\|\hat{x}^{(j)} - w_j^o\| \leqslant \sqrt{M}\|[\epsilon]_j\|$, where $[\epsilon]_j$ and w_j^o represent the jth columns of $[\epsilon_1, \epsilon_2, 0]^T$ and $[W_1^o, W_2^o]^T$, respectively. This implies that $\|\hat{x} - [W_1^o, W_2^o]^T\| \leqslant \sqrt{M}\|\epsilon\|$, where $\hat{x} = [\hat{x}_1, \cdots, \hat{x}_m]$ and $M = \kappa([\tilde{X}^{(j)}]^T\tilde{X}^{(j)})$. Note that

$$[\tilde{X}^{(j)}]^T\tilde{X}^{(j)} = \begin{pmatrix} H_1H_1^T + \lambda\text{diag}(E_j) & -\lambda\text{diag}(E_j) \\ -\lambda\text{diag}(E_j) & H_2H_2^T + \lambda\text{diag}(E_j) \end{pmatrix}.$$

We complete the proof of the corollary.

□

Because $\tilde{X}^{(j)}$ defined in the proof of Corollary 1 has a uniformly bounded condition number κ over j due to the fact that E_j's are bounded, Theorem 1 implies that $\hat{W}_1, \hat{W}_2$ are as closed as the norm of the error to the underlying W_1^o, W_2^o. Corollary 1 also shows that the proposed estimator with an appropriately chosen ζ_n has the bounded estimation error. The condition imposed on ζ_n implies that the weight E_{jk} is non-zero only when the underlying parameter satisfies $[W_0^{(1)}]_{jk}^T = [W_0^{(2)}]_{jk}^T$. Although the condition imposed on ζ_n can not be verified in real data application, in theory, this condition always holds for ζ_n in an appropriate range.

S3.2 Sensitivity analysis regarding to ζ_n

We performed simulations to study the sensitivity of the results with respect to ζ_n. We used the following model:

$$Y_1 = W_1H_1, \quad Y_2 = W_2H_2, \tag{S1}$$

In this model, data were simulated under $n = 200$, $m = 50$, and $k = 5$. All columns in H_1 were generated from the same Dirichlet distribution, while all columns in H_2 were from another Dirichlet distribution, i.e., $\vec{h}_i^1 \overset{\text{i.i.d.}}{\sim} Dir(\pi_1)$ and $\vec{h}_i^2 \overset{\text{i.i.d.}}{\sim} Dir(\pi_2)$. For the concentration parameters π_k, we used $\pi_1 = \pi_2 = [1, 2, 3, 4, 5]$.For W_1 and W_2, we constructed a matrix W with i.i.d. N(0,2) entries, then randomly chose 5% entries, called Ω, such that the selected genes were differentially expressed for the two groups. Then, we set $[W_1]_{ij} = [W_2]_{ij} = W_{ij}$ for $(i,j) \notin \Omega$, and $[W_1]_{ij} = 0.5W_{ij}$ and $[W_1]_{ij} = 2W_{ij}$ for $(i,j) \in \Omega$.For the ζ_n value, we considered $\zeta_n \in \{1, 2, 4, 8, 16, 32, 64, 128, 256, 512\}$.

Tables S1 and S2 summarize the mean correlations of the estimated H_k from different ζ_n for $k = 1, 2$, respectively. We can see that the obtained H_ks were

quite robust with respect to ζ_n values, demonstrating the robustness of the result when ζ_n is in an appropriate range. Tables S3 and S4 show the relative difference norm of the estimate W_k obtained from the different ζ_n for $k = 1, 2$, respectively. For example, for the estimates obtained from ζ_1 and ζ_2, we record $\|W_k(\zeta_1) - W_k(\zeta_2)\|/(\|W_k(\zeta_1)\| + \|W_k(\zeta_2)\|)$, where $W_k(\zeta)$ is the output using the ζ value. We can observe that the relative errors are quite small, which implies that the results of W_ks are robust to the choice of ζ_n if it is chosen from an appropriate range.

ζ_n	1	2	4	8	16	32	64	128	256	512	1024
1	1.000	0.998	0.998	0.998	0.998	0.998	0.996	0.996	0.994	0.990	0.994
2	0.998	1.000	1.000	1.000	1.000	1.000	0.998	0.998	0.996	0.992	0.996
4	0.998	1.000	1.000	1.000	1.000	1.000	0.998	0.998	0.996	0.992	0.996
8	0.998	1.000	1.000	1.000	1.000	1.000	0.998	0.998	0.996	0.992	0.996
16	0.998	1.000	1.000	1.000	1.000	1.000	0.998	0.998	0.996	0.992	0.996
32	0.998	1.000	1.000	1.000	1.000	1.000	0.998	0.998	0.996	0.992	0.996
64	0.996	0.998	0.998	0.998	0.998	0.998	1.000	1.000	0.998	0.994	0.998
128	0.996	0.998	0.998	0.998	0.998	0.998	1.000	1.000	0.998	0.994	0.998
256	0.994	0.996	0.996	0.996	0.996	0.996	0.998	0.998	1.000	0.996	1.000
512	0.990	0.992	0.992	0.992	0.992	0.992	0.994	0.994	0.996	1.000	0.996
1024	0.994	0.996	0.996	0.996	0.996	0.996	0.998	0.998	1.000	0.996	1.000

Table S1: Correlation of the estimate H_1 obtained from different ζ_n values.

ζ_n	1	2	4	8	16	32	64	128	256	512	1024
1	1.000	1.000	1.000	1.000	1.000	1.000	0.998	0.998	0.994	0.990	0.992
2	1.000	1.000	1.000	1.000	1.000	1.000	0.998	0.998	0.994	0.990	0.992
4	1.000	1.000	1.000	1.000	1.000	1.000	0.998	0.998	0.994	0.990	0.992
8	1.000	1.000	1.000	1.000	1.000	1.000	0.998	0.998	0.994	0.990	0.992
16	1.000	1.000	1.000	1.000	1.000	1.000	0.998	0.998	0.994	0.990	0.992
32	1.000	1.000	1.000	1.000	1.000	1.000	0.998	0.998	0.994	0.990	0.992
64	0.998	0.998	0.998	0.998	0.998	0.998	1.000	1.000	0.996	0.992	0.994
128	0.998	0.998	0.998	0.998	0.998	0.998	1.000	1.000	0.996	0.992	0.994
256	0.994	0.994	0.994	0.994	0.994	0.994	0.996	0.996	1.000	0.992	0.994
512	0.990	0.990	0.990	0.990	0.990	0.990	0.992	0.992	0.992	1.000	0.998
1024	0.992	0.992	0.992	0.992	0.992	0.992	0.994	0.994	0.994	0.998	1.000

Table S2: Correlation of the estimate H_2 obtained from different ζ_n values.

ζ_n	1	2	4	8	16	32	64	128	256	512	1024
1	0.000	0.000	0.000	0.000	0.002	0.003	0.006	0.008	0.012	0.016	0.016
2	0.000	0.000	0.000	0.000	0.002	0.003	0.006	0.008	0.012	0.016	0.016
4	0.000	0.000	0.000	0.000	0.002	0.003	0.006	0.008	0.012	0.016	0.016
8	0.000	0.000	0.000	0.000	0.001	0.003	0.006	0.008	0.011	0.016	0.016
16	0.002	0.002	0.002	0.001	0.000	0.001	0.004	0.007	0.010	0.015	0.015
32	0.003	0.003	0.003	0.003	0.001	0.000	0.003	0.006	0.010	0.014	0.015
64	0.006	0.006	0.006	0.006	0.004	0.003	0.000	0.003	0.007	0.011	0.013
128	0.008	0.008	0.008	0.008	0.007	0.006	0.003	0.000	0.004	0.009	0.011
256	0.012	0.012	0.012	0.011	0.010	0.010	0.007	0.004	0.000	0.005	0.008
512	0.016	0.016	0.016	0.016	0.015	0.014	0.011	0.009	0.005	0.000	0.009
1024	0.016	0.016	0.016	0.016	0.015	0.015	0.013	0.011	0.008	0.009	0.000

Table S3: Relatively difference between W_1s obtained from different ζ_n values.

ζ_n	1	2	4	8	16	32	64	128	256	512	1024
1	0.000	0.000	0.000	0.000	0.001	0.002	0.005	0.009	0.015	0.021	0.025
2	0.000	0.000	0.000	0.000	0.001	0.002	0.005	0.009	0.015	0.021	0.025
4	0.000	0.000	0.000	0.000	0.001	0.002	0.005	0.009	0.015	0.020	0.025
8	0.000	0.000	0.000	0.000	0.001	0.002	0.005	0.009	0.015	0.020	0.025
16	0.001	0.001	0.001	0.001	0.000	0.001	0.004	0.008	0.014	0.020	0.024
32	0.002	0.002	0.002	0.002	0.001	0.000	0.003	0.008	0.014	0.019	0.024
64	0.005	0.005	0.005	0.005	0.004	0.003	0.000	0.004	0.010	0.016	0.020
128	0.009	0.009	0.009	0.009	0.008	0.008	0.004	0.000	0.006	0.012	0.016
256	0.015	0.015	0.015	0.015	0.014	0.014	0.010	0.006	0.000	0.006	0.011
512	0.021	0.021	0.020	0.020	0.020	0.019	0.016	0.012	0.006	0.000	0.006
1024	0.025	0.025	0.025	0.025	0.024	0.024	0.020	0.016	0.011	0.006	0.000

Table S4: Relatively difference between W_2s obtained from different ζ_n values.

Next, we examine how ζ_n might affect real data DEG outcomes. We ran SCADIE with $\zeta_n = 2$ and $\zeta_n = 0.1$, and plot volcano plots similar to Figure 5. As can be seen from Figure S7, the DEGs log2 fold-change and p-values are largely similar, which indicates our DEG results are robust if the parameter ζ_n is in the appropriate range.

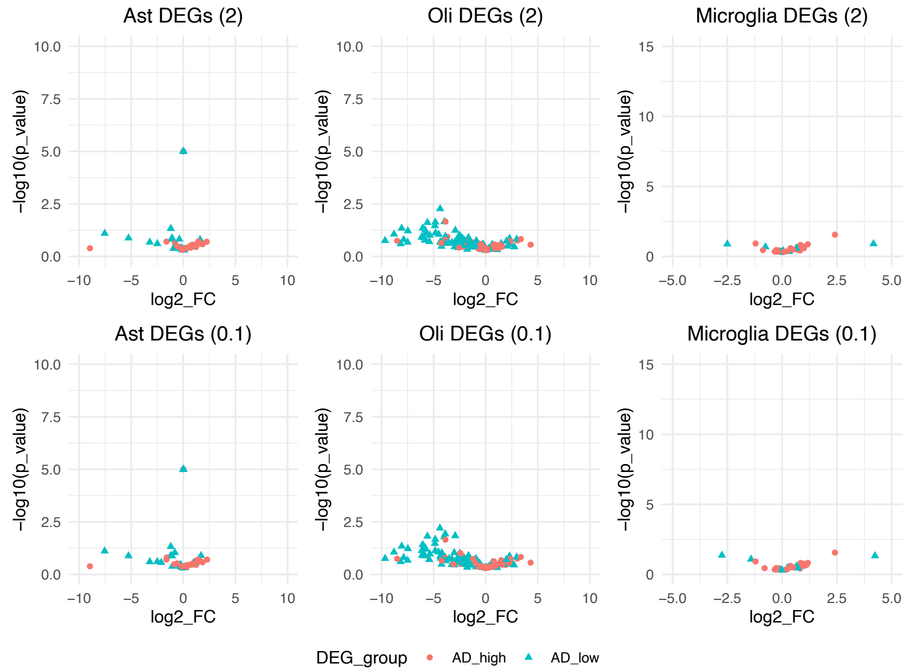

Figure S7: Volcano plots for cell type-specific DEGs in AD under different ζ_ns.

S3.3 Computational efficiency

We conducted simulations to investigate the computational cost of the proposed algorithm. We used the following model:

$$Y_1 = W_1 H_1, \quad Y_2 = W_2 H_2.$$

To assess whether the proposed algorithm is even feasible to be run on the whole transcriptome, we set $m \in \{500, 1000, 2000, 5000, 10000\}$, $n = 100$, and $k = 5$ to simulate data. The other model parameters are set as in Subsection S3.2. We conduct simulation 50 times and record its average minutes and the one standard deviations. We compare the proposed SCADIE with the conventional NMF method, which updates W_1 and W_2 separately via NNLS. As can be seen in Table S5, compared to the NNLS, SCADIE takes more time. But even for large $m = 10000$ case, the proposed algorithm takes less than 8 minutes on average, which implies that it may be feasible in such large data cases.

Method	m				
	500	1000	2000	5000	10000
SCADIE	0.4 (0.1)	0.7 (0.3)	1.9 (0.5)	4.4 (0.9)	7.8 (1.2)
NNLS	0.2 (0.1)	0.3 (0.1)	0.7 (0.3)	2.0 (0.5)	3.6 (0.8)

Table S5: Average of computational time and 1 standard deviation in parenthesis obtained from different n values. It is implemented on a linux server (Intel Server System R2308WF (2U / Xeon 6226R x2CPU / 768GB Memory)) using the R.

S3.4 Performances of SCADIE under different cell type-sample size ratios

As discussed in Section "DEG identification under poor initialization and limited sample size", SCADIE's performance might be limited when the sample size only marginally larger than the number of cell types. In this section we perform a comprehensive examination of sample size's effect on outcome quality through simulations.

We first conducted simulations to investigate how estimation performance of SCADIE depends on the underlying number of cell-types compared to the sample sizes. Given the number of samples n and the number of genes m, we assume that the underlying number of cell types k is less than $\min(n, m)$. This is because if $k \geqslant \min(n, m)$, the columns of the factor matrix W or F is linearly dependent or full rank, which is undesired case in the factorization methods such as NMF. Note that one of the main goals of NMF is to reduce the number of parameters in the estimation procedure using $k(n + m)$ parameters. If $k \geqslant \min(n, m)$, the number of estimated parameters is $k(n + m) \gtrsim nm$, which is the order of the number of components in the data Y.

Thus, in this simulation, for a given underlying number of cell types $k = 5$ and the number of genes 200, we consider $n \in \{6, 10, 20, 50, 100, 200, 500, 1000\}$ number of samples in SCADIE. The other model parameters are set as in Subsection S3.2. For the estimates $\hat{W}_k$ and $\hat{H}_k$, we record the relative error (RE) of the $\hat{W}_k$ with respect to the underlying W_k, i.e., $\|\hat{W}_k - W_k\|_F / \|W_k\|_F$, and the average correlation of columns of $\hat{H}_k$ and H_k. Table S6 reports the performances of SCADIE when the sample size n varies. It can be seen that SCADIE's output Ws and Hs show robust high similarities with groundtruth when sample size equal or greater than 2x number of cell types.

n	6	10	20	50	100	200	500	1000
RE ($\hat{W}_1$)	0.26	0.18	0.18	0.18	0.12	0.16	0.19	0.12
RE ($\hat{W}_2$)	0.30	0.22	0.19	0.18	0.19	0.15	0.18	0.12
Correlation ($\hat{H}_1$)	0.79	0.86	0.84	0.85	0.82	0.85	0.85	0.90
Correlation ($\hat{H}_2$)	0.81	0.89	0.87	0.85	0.83	0.90	0.87	0.92

Table S6: Performances of the estimates with respect to sample sizes.

To further investigate how these similarities reflect on DEG outcomes, we used the previous mouse ISC pseudo bulk data set to benchmark DEG identifications. There are four cell types in the dataset and we used sample sizes equal 6, 8, 12, 16, 20 in our simulations.

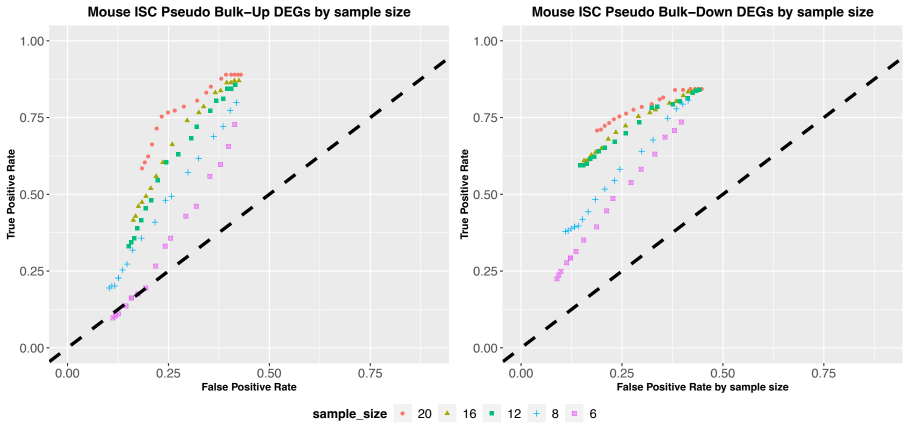

Figure S8: Sensitivities and false positive rates for up-regulated and down-regulated DEGs over a range p values under different sample sizes

As can be seen from Figure S8, both sensitivity and specificity increase with sample size, and when sample size equals 6, SCADIE's performance in up-regulated DEGs was not significantly better than random. Taken together, we recommend using SCADIE for DEG identification when sample size is at least 1.5x of number of cell types.

S3.5 When the sample sizes n_1 and n_2 are different

SCADIE gives the same weight to the first two terms in the objective function, i.e., $w_1 = w_2$ in $w_1\|Y_1 - W_1H_1\|_F^2 + w_2\|Y_1 - W_1H_1\|_F^2$. One may take different values to w_1 and w_2, e.g., depending on the sample sizes, one may set $w_1/w_2 = n_2/n_1$. Specifically, we propose to use $w_1 = n2/n1$, $w_2 = 1$ for the first two terms if the ratio of n_1 and $n2$ is very different from 1. This is

motivated by the fact that the first two terms can be understood as empirical risks, by averaging the loss function on the samples.

In this subsection, we conduct simulation analysis to investigate the performance of SCADIE and the weighted SCADIE by assigning different weights on the first two terms such that $w_1 = n2/n1$, $w_2 = 1$, when the ratio of the sample sizes of two groups vary. We set the number of samples $n_1 = 100$ and $n_2 = [\alpha n_1]$, where $\alpha \in \{\frac{1}{50}, \frac{1}{20}, \frac{1}{10}, \frac{1}{4}, \frac{1}{2}, 1, 2, 4, 10, 20, 50\}$.

The other model parameters are set as in Subsection S3.2. We conduct simulation 50 times and record average performances of SCADIE and the weighted SCADIE using the measures defined in Subsection S3.4. As can be seen in Table S7, for all the ratio values α, SCADIE well estimates H_i and W_i.

Table S8 records the performance measures of the weighted SCADIE, which shows that weighted SCAIDE performs better than SCADIE when the ratio of two dimensions are very small or large, e.g., $\alpha \in \{\frac{1}{50}, 50\}$. However, when the ratio of two dimensions are moderate, the weighted SCADIE does not outperform SCADIE. Given that ratios of sample sizes in our real data analyses always between 1/10 and 10, assigning equal weights to the first two terms in the objective function in SCADIE seems to be reasonable.

It is worth noting that imposing another tuning parameter to the first or second term can improve the performances of SCADIE, but it will take additional time to tune the additional tuning parameter. We leave this issue for future research.

α	$\frac{1}{50}$	$\frac{1}{20}$	$\frac{1}{10}$	$\frac{1}{4}$	$\frac{1}{2}$	1	2	4	10	20	50
RE ($\hat{W}_1$)	0.20 (0.18)	0.15 (0.05)	0.20 (0.21)	0.16 (0.15)	0.17 (0.16)	0.19 (0.14)	0.13 (0.08)	0.12 (0.06)	0.16 (0.14)	0.19 (0.20)	0.14 (0.11)
RE ($\hat{W}_2$)	0.20 (0.18)	0.21 (0.09)	0.23 (0.22)	0.17 (0.16)	0.18 (0.17)	0.19 (0.15)	0.11 (0.06)	0.13 (0.06)	0.16 (0.14)	0.18 (0.15)	0.19 (0.24)
Correlation ($\hat{H}_1$)	0.84 (0.19)	0.87 (0.06)	0.83 (0.20)	0.85 (0.18)	0.86 (0.17)	0.82 (0.17)	0.91 (0.05)	0.91 (0.04)	0.88 (0.09)	0.87 (0.14)	0.85 (0.18)
Correlation ($\hat{H}_2$)	0.80 (0.28)	0.88 (0.09)	0.82 (0.20)	0.87 (0.12)	0.86 (0.22)	0.83 (0.20)	0.93 (0.04)	0.91 (0.04)	0.89 (0.11)	0.86 (0.18)	0.85 (0.24)

Table S7: Average (one standard deviation) of the performance measures of SCADIE with respect to ratio of sample sizes.

α	$\frac{1}{50}$	$\frac{1}{20}$	$\frac{1}{10}$	$\frac{1}{4}$	$\frac{1}{2}$	1	2	4	10	20	50
RE ($\hat{W}_1$)	0.18 (0.12)	0.14 (0.05)	0.15 (0.06)	0.15 (0.10)	0.15 (0.13)	0.19 (0.14)	0.20 (0.17)	0.18 (0.14)	0.18 (0.13)	0.19 (0.08)	0.15 (0.12)
RE ($\hat{W}_2$)	0.18 (0.15)	0.19 (0.10)	0.17 (0.09)	0.16 (0.10)	0.19 (0.14)	0.19 (0.15)	0.20 (0.18)	0.18 (0.15)	0.17 (0.11)	0.18 (0.10)	0.15 (0.13)
Correlation ($\hat{H}_1$)	0.88 (0.14)	0.88 (0.18)	0.88 (0.06)	0.86 (0.12)	0.86 (0.16)	0.82 (0.17)	0.83 (0.12)	0.86 (0.11)	0.83 (0.17)	0.86 (0.10)	0.85 (0.15)
Correlation ($\hat{H}_2$)	0.83 (0.22)	0.89 (0.20)	0.87 (0.08)	0.90 (0.08)	0.87 (0.15)	0.83 (0.20)	0.84 (0.19)	0.89 (0.12)	0.86 (0.14)	0.87 (0.21)	0.87 (0.15)

Table S8: Average (one standard deviation) of the performance measures of the weighted SCADIE with respect to ratio of sample sizes.

S3.6 Comparison of Jackknife and Bootstrap

We evaluated the jackknife standard error estimates by comparing them with bootstrap estimates on the simulated data described in Section "Methods - Simulation models and benchmarking" in the main text.

For bootstrapping, we chose a wide range for the number of resamplings: from half of sample size 10, to 20x sample size 400. And each random sample was generated by sampling 20 columns (which equals the observed sample size) from Y_1, Y_2 and H_1, H_2, respectively. And the standard error $\Sigma^{bs}_{W_1-W_2}$ was obtained by empirical standard error across all samples.

The column-column correlations between jackknife $\Sigma_{W_1-W_2}$ and bootstrap $\Sigma^{bs}_{W_1-W_2}$s show extremely high concordances (Figure S9).

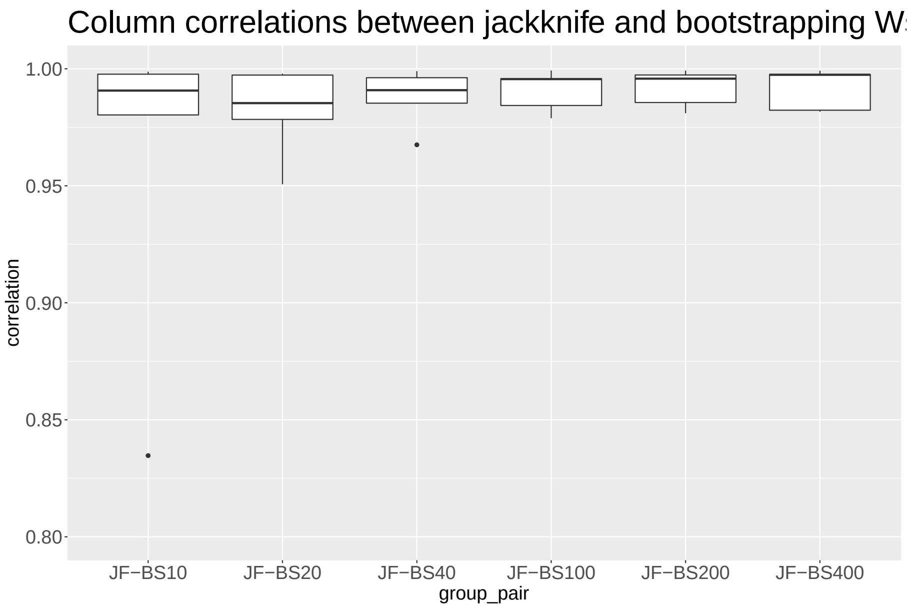

Figure S9: JF: jackknife; BS10: bootstrapping with the number of resamplings equals 10; BS20: bootstrapping with the number of resamplings equals 20; BS40: bootstrapping with the number of resamplings equals 40; BS100: bootstrapping with the number of resamplings equals 100; BS200: bootstrapping with the number of resamplings equals 200; BS400: bootstrapping with the number of resamplings equals 400.

Next, we investigate the run time of jackknife and bootstrap. As can be seen from Figure S10, the run time is proportional to the number of SCADIE runs.

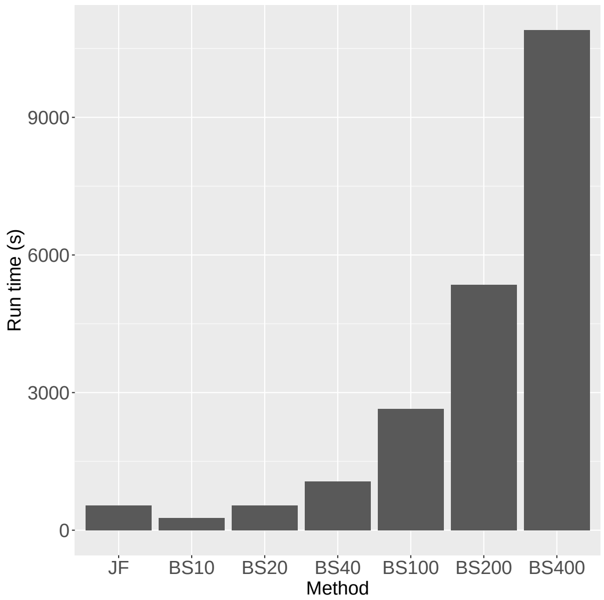

Figure S10: Run time for jackknife and bootstrap with different numbers of re-sampling.

Although jackknife and bootstrap show highly concordant empirical results, due to the sampling with replacement nature of bootstrap, there exists risk of higher noise due to singularity in Ys and Hs, i.e., the duplicated columns in sampled Y^{sample} and H^{sample} increases uncertainty in regression. In this regard, we recommend using jackknife for general purposes. In general, the bootstrap is more computationally intensive than Jackknife [35]. The Jackknife tends to perform better for confidence interval estimation for pairwise agreement measures and the Jackknife is more suitable for small original data samples [6].
