# Supplementary material for: SCADIE: simultaneous estimation of cell type proportions and cell type-specific gene expressions using SCAD-based iterative estimating procedure
Source: Genome Biol. 2022 Jun 15;23:129. doi: 10.1186/s13059-022-02688-w (PMC9199219; doi:10.1186/s13059-022-02688-w)
Supplement: Supplementary file 3 — Additional file 3 Review history. [file 13059_2022_2688_MOESM3_ESM.docx]

**1^st^ round**

**Reviewer 1**

Tang and coauthors proposed SCADIE, a method for simultaneous estimation of cell type proportions and cell type-specific gene expressions. While such a method is certainly welcome to the field, major revisions are needed for it to be suited for publication in Genome Biology.

Overall, the writing of the paper can be significantly improved.

1) The Introduction contains several inaccurate references to existing methods. For example, "More specifically, these deconvolution methods curate a signature matrix with only a subset of cell type-specific genes." This is not true, as several recently developed deconvolution methods use all genes (with different weights). "Although the applications of these deconvolution methods have led to many findings, they implicitly attribute the observed differences in bulk gene expression levels to cell type proportion differences across samples." This is also a very inaccurate statement. One of the earliest methods, csSAM, performs case-control testing after deconvolution and certainly does not make this assumption.

2) From the introduction, "SCADIE is built on the assumption that most genes in W should be similar between the two groups of samples, which likely holds in many practical settings." This is too strong a statement that does not hold. As a matter of fact, SCADIE carries out testing on the difference between W under different conditions, and I find this is somehow contradictory to the statement. Note that I am not saying that the assumption is invalid (since it only applies to the initialization and will be later updated), but rather the writing can be improved to get the actual underlying assumption described correctly.

3) In the Introduction, it would be good to let the readers know what experimental design/data input SCADIE would be applied to. This is not clear until I read the later sections. I thought SCADIE would need scRNA-seq for both case and control, but this is not the case. On this note, how would SCADIE be adapted to limited case-control scRNA-seq data on a limited number of individuals? Would this be helpful for the initialization?

4) A lot of the short paragraphs contain a sentence of two. For example, the section "DEG identification under poor initialization and limited sample size" on pages 13-14. Also, the paper contains several bulletin points and lists within the main manuscript. This is rarely seen in published journal articles.

5) On page 5, some high-level introduction to the SCAD function is needed - this is the key methodological contribution of this paper, and SCAD is also in the title. So some background is certainly needed.

6) For the section "Robustness with respect to Zeta_n", Zeta_n is not even introduced in the main manuscript, and this can be confusing to the readers.

I am concerned with the false positives and false negatives of the methods. For the example shown in Figure 5, what's the difference between the two columns in table f, "the percentages that were significant from SCADIE output" and "the percentages that were correctly significant from SCADIE?" The difference is quite dramatic - are they called to be in different directions by SCADIE vs by single-cell case-control data? Also, for the thousands of the additional DE genes called by SCADIE, the authors picked the top 42 DE genes unique to SCADIE, and identified 19 to be associated with COPD and 33 to have the same directional change. What about the rest of the genes? A geneset enrichment analysis on the genes in the last column of table f? Similarly, for the boxplot in Figure 8, the boxplot of known DEGs was only marginally significant for the known up-regulated genes, but not for the down-regulated genes. Are these false negatives?

Since SCADIE adopts a SCAD-based function as well as NNLS during the iterations, the computational efficiency needs to be assessed. Is it even feasible to be run on the whole transcriptome?

Similar to csSAM, does SCADIE perform a joint test between W and H? Also, an omnibus test across cell types?

For the objective functions shown on pages 17 and 18, the first two terms are given equal weights, but their dimensions can differ. A similar framework (albeit on different types of data) https://doi.org/10.1073/pnas.1805681115 gives another tuning parameter to the second term.

**Reviewer 2**

The authors proposed the SCADIE approach, an iterative algorithm that can simultaneously estimate cell type-specific gene expression profiles and cell type proportions, and perform cell type-specific differential expression analysis at the group level. The work is interesting with a few novel ideas particularly the Lasso-type joint learning strategy, and may complement many existing supervised deconvolution methods. However, there are some major concerns that need to be carefully addressed and some conclusions may be adjusted.

My specific critiques are:

1) Methods for computational deconvolution may be divided into supervised and unsupervised categories with a few exceptions of semi-supervised or sample-level approaches. To my understanding, SCADIE is a supervised population-level deconvolution method with unique focus on two group differential analysis. This nature should be clarified clearly.

2) The inputs required and assumptions made by SCADIE should be explicitly explained, e.g., proportion matrix or common signature matrix, and assumed known yet common number of cell types within/across groups. It is unclear whether the number and identify of cell types across two groups are assumed to be known and the same. For some disease lesions, some cell types are significantly altered, for example, in tumor or anthracnosis, involving novel cell subtypes that have no reference to be used (e.g. due to novel mutations). The authors should discuss these issues and clarify the applicability of SCADIE in a broad context.

3) In terms of biological diversity, some cell types are made up of complex mixtures rather than simple or known cell types. Microenvironment, cell-cell interaction, and developmental maturity are a few examples of situations when the subtypes are difficult to define. While cells can participate in various biological functions, we may need to acknowledge that one cell may fit into different subtype categories based on different classification methods, and they are all valid subtypes. There are several unsupervised deconvolution methods which have been proposed and some are readily accessible thus should not be completely disregarded (Hart, Sheftel et al. 2015, Chen, Wu et al. 2020). The authors may want to give a brief overview on this alternative.

4) Some statements are either unclear or inaccurate and should be corrected, e.g., "It is easy to see that directly estimating W and H without any constraint is not an identifiable problem.", "most deconvolution methods only focus on a subset of cell type-specific genes (i.e. signature genes), while all the genes in the genome are of interests in DEG analyses." Does 'simultaneously/directly' imply supervised or unsupervised approach? Many deconvolution methods are applicable to deconvoluting all genes once the model is estimated.

5) The selection of K-L divergence to measure H accuracy needs some justification. Will such a measure be dominated by the abundant cell types?

6) In Figure 1, it is unclear whether cell type proportion matrix indicated in the text or signature matrix shown in the figure is required by SCADIE. Please clarify.

7) Please clarify 'point estimates', 'entry-wise', etc.

8) Please explain what 'ROC-like curve' is and how it was generated. It is unclear why the ROC-like curves of the two peer methods are short and 'separated'. How were the ground truth DEGs specified?

9) The statement 'It should be noted that since CIBERSORTx' high resolution mode is only able to recover a small fraction of Ws, its overall sensitivity is limited.' Needs to be explained.

10) Regarding the advantages of SCADIE (actually two aspects), the statement was not clear and should be revisited. Also, the text 'In terms of functionality, SCADIE is the first computational method that can estimate and perform cell type-specific differential expression analysis at whole transcriptome level, provide matched cell type proportion estimates, while working with multiple types of expression data.' is an overstatement and should be adjusted or specified.

Hart, Y., H. Sheftel, J. Hausser, P. Szekely, N. B. Ben-Moshe, Y. Korem, A. Tendler, A. E. Mayo and U. Alon (2015). "Inferring biological tasks using Pareto analysis of high-dimensional data." Nat Methods 12(3): 233-235.

Chen, L., C. T. Wu, N. Wang, D. M. Herrington, R. Clarke and Y. Wang (2020). "debCAM: a bioconductor R package for fully unsupervised deconvolution of complex tissues." Bioinformatics 36(12): 3927-3929.

**Reviewer 3**

The research tries to bring an iterative algorithm to simultaneously address the estimation of cell-type proportions and cell type-specific gene expression in bulk sequencing data. Many existing deconvolution methods focused only on the cell type proportion estimation with the assumption of a fixed cell type-specific expression. Many differential expression genes analysis methods raised DEGs without the elimination of effects from diversified cell-type proportions. SCADIE, however, updates the two estimations together, offers more confidence on group-specific DEGs analysis. Also, compared to the existed deconvolution method, like CIBERSORTx, instead of working on a small signature matrix, SCADIE can work at the whole transcriptome level, thus offering more potential DEG candidates.

SCADIE takes the combination of iterative NNLS optimization algorithms, SCAD-penalty, and jackknife method to carry out the whole estimation procedures. The highlighted part of the method lies in the SCAD penalty between cell type-specific expressions from two groups. Unlike the traditional penalization methods L1 or L2 penalties, the SCAD-penalty can set bounded regularization for the difference within a small range and at the same time penalize less on the large difference compared to L1/L2. This theoretically can help to regularize on the dissimilarity for cell type-specific expression in the two groups while at the same time maintain the true differences in the two groups. Thus, it carries out similarly to the practical cases.

The paper is well written. The results and methods are quite clear and easy to understand. The mathematical part for the methods is also well-calibrated. The author also offers very detailed comparisons on a group of datasets including simulation, pseudo-bulk, and real bulk samples). However, when it comes to the results part, some of the results may be less convincing to support their claimed advantages compared to the existed methods.

Few comments for the results and methods in the paper:

Results:

Cell-type proportions estimations:

For cell-type proportions estimations, SCADIE benchmarked against DWLS, CIBERSORTx, and naïve NNLS. Given the pseudo-bulk dataset has the single-cell reference data, and MuSiC is also a state-of-art method, a comparison including MuSiC would be more convincing. (Page 3, line 47)

The evaluated metric for cell-type proportions is K-L divergence in this manuscript. For benchmark papers in the deconvolution area, the standard and stringent metrics are mostly correlation, mean absolute deviance, or RMSE. (Page 3, line 56)

In supplementary figure S3a. The performance of SCADIE is not significantly better compared to the other methods. Raising the question of improvement on the accuracy of H (cell type proportions) over iterative estimations.

SCAIE can better identify DEGs:

For DEG analysis, before simultaneous estimation, we can estimate W (cell type-specific expression) from the proportions calculated from devolution tools first, and then carried out DEG analysis for two groups. Here in Figure2, only NNLS, csSAM, CIBERSORTx are included. And csSAM is published for microarray deconvolution ten years ago. Including the W estimated from MuSiC etc. and include their DEG analysis would be more convincing. (Page 5, line 5)

In Figure2, the DEG identification performance of SCADIE is not that significant as the author has claimed. In most cases, the performance overlapped with NNLS, while in the cases where the FPR decreases the TPR also decreases (except for one case, Mouse ISC Pseudo Bulk1 -Up DEGs). This can raise from the decrease in the number of DEGs detected. (Page 5, line 60; Page 6, line 8)

Improve the estimates from other methods

In Figure3, the improvement for accuracy with the iterative procedure is not that clear. For many cases, with a decrease or increase in both FPR and TPR.

SCADIE's performance on real data (COPD)

Figure4 offers a clear cell-type proportions structure. And carry out the large epithelial proportion variation for COPD groups, which is consistent well with the literature.

In Figure5, 60% of single-cell identified DEGs showed just concordant directional changes from SCADIE output, and only 12-48% of single-cell identified DEGs were inferred to be DEGs by SCADIE. The percentages are not that strong. So, the claim of good performance might need support from the comparison with other methods. (Page 7, line 60)

Literature review to find the associations between SCADIE-unique genes and COPD better testify the signals detected from SCADIE. (Page 9, line 12)

SCADIE's performance on real data (AD)

In Figure7, after the SCADIE procedure, the ratios of known DEGs that are estimated to have fold-changes in the opposite directions decreases. However, the p-values and fold-changes values are also sacrificed, with more DEGs fall into the insignificant regions. (Page 11, line 9)

DEG identification under poor initialization and limited sample size

The results may depend on the initial results of estimated cell-type proportions from other deconvolution methods, as well as the quality of the data. (Page 11, line 47)

How to determine the maximum number of cell types the SCADIE can deal with is not mentioned. Should be discussed for the user to have a better idea about the cell type numbers they can apply with the given sample number. (Page 11, line 62)

In Figure8, the SCADIE final W in 8a, though identified more up-regulated and down-regulated DEGs, but also more down-regulated DEGs detected as up-regulated. And the log2-expression fold change in 8b, though the p-values decrease compared to the CIBERSORTx, they are still insignificant, which proves again the SCADIE's estimation performance depends heavily on the correct initialization and enough sample size. (Page 12, line 47-54)

Methods:

They build the whole algorithm with the assumption that most genes should have a proper dissimilarity level between the two groups of samples, which resembles many practical settings, also support the theoretical proposal for the selected penalty. (Page 2, line 46)

The two highlighted novel strategies for SCADIE are:

The selection of a SCAD-derivative based penalty function (Page 3, line 30)

A parallel leave-one-out jackknife procedure to obtain the standard error for cell type-specific expression difference between the two groups. This can ease the further DEG analysis. (Page 3, line 37)

Given DEG analysis is one of the key advantages the author tried to highlight in many sections. The hypothesis testing procedures might need a more detailed description in the manuscript. (Page 3, line 42).

The reasonable range is not clearly presented. (Page 6, line 60)

The selected value for is not clearly discussed in the paper. (Page 15, line 28)

Might change a to to keep consistency. (Page 16, line 18-22)

Given jackknife standard estimation is one of the key highlights in SCADIE, its advantage over bootstrap's sampling is not clearly presented. For the results provided in Supplementary section S3.3, these two methods have a high concordance. The potential singularity issue is not well explained and presented. (Page 16, line 61)

**Authors’ response**

We would like to first thank you for all the insightful and constructive comments and apologize for the late turnaround. Those useful suggestions have led us to rethink many aspects of our manuscript, from analyses to results presentation. In this revision, we have redone some previous analyses and included several additional ones to better examine SCADIE’s performance under various conditions. In addition, we have made extensive changes to our manuscript in terms of both figures and text, to improve its comprehensiveness and accuracy. Below is a summary of major changes made:

1.More in-depth examinations of SCADIE’s advantages over existing methods: to further evaluate SCADIE’s advantages, we have included benchmarking with two additional methods, MuSiC and TOAST, in terms of cell proportion estimates and cell type specific DEG identifications. For real data analysis, we performed several additional analyses, including GSEA, to provide a more objective examination on SCADIE’s capacity and potential false positive issues.

2.Including results using more consistent benchmarking metrics: in addition to our previous analyses use K-L divergence as a metric that we think has certain advantages, we have added benchmarking results using RMSE as another metric. Overall results measured by these two metrics are highly consistent.

3.Additional analyses and discussion on the applicability of SCADIE in various conditions: several comments have made us realize that it is important to better understand SCADIE’s performance and efficiency in varying conditions, including uneven or limited sample sizes, poor initialization, and others. To this end, we have included additional simulations to evaluate SCADIE’s accuracy and efficiency in those conditions, and provided more detailed discussion on its applicability in the revised manuscript.

4.Improved writing, figure, and table presentations: many comments on the previous manuscript focused on the comprehensiveness of this paper. To this end, we have re- written a substantial portion of our manuscript to improve the paper’s readability, these include a clearer description of SCADIE’s input and procedure, fixing of many inaccurate and over statements, re-arrangements of certain tables/figures, and removal of unnecessary bullet points.

On top of the above four major aspects, many other minor changes have been made.

Please kindly refer to the point-by-point response below for more comprehensive explanations. We do hope these changes can address all your previous concerns, and we are very glad to answer should there be additional questions from you.

Thank you all very much, and we look forward to hearing back from you!

Daiwei Tang, Seyoung Park, and Hongyu Zhao

Reviewer #1: Tang and coauthors proposed SCADIE, a method for simultaneous estimation of cell type proportions and cell type-specific gene expressions. While such a method is certainly welcome to the field, major revisions are needed for it to be suited for publication in Genome Biology. Overall, the writing of the paper can be significantly improved.

1) The Introduction contains several inaccurate references to existing methods. For example, "More specifically, these deconvolution methods curate a signature matrix with only a subset of cell type-specific genes." This is not true, as several recently developed deconvolution methods use all genes (with different weights). "Although the applications of these deconvolution methods have led to many findings, they implicitly attribute the observed differences in bulk gene expression levels to cell type proportion differences across samples." This is also a very inaccurate statement. One of the earliest methods, csSAM, performs case- control testing after deconvolution and certainly does not make this assumption.

Response: Thank you for pointing out these inaccurate statements in our previous manuscript. We have re-written these paragraphs to ensure a more accurate reference to the existing methods.

2) From the introduction, "SCADIE is built on the assumption that most genes in W should be similar between the two groups of samples, which likely holds in many practical settings." This is too strong a statement that does not hold. As a matter of fact, SCADIE carries out testing on the difference between W under different conditions, and I find this is somehow contradictory to the statement. Note that I am not saying that the assumption is invalid (since it only applies to the initialization and will be later updated), but rather the writing can be improved to get the actual underlying assumption described correctly.

Response: Thank you for this correction. We have re-written most of the introduction section, including this corresponding statement, to express the principle behind SCADIE more accurately (Page 3 line 5-13, 21-29).

3) In the Introduction, it would be good to let the readers know what experimental design/data input SCADIE would be applied to. This is not clear until I read the later sections. I thought SCADIE would need scRNA-seq for both case and control, but this is not the case. On this note, how would SCADIE be adapted to limited case-control scRNA-seq data on a limited number of individuals? Would this be helpful for the initialization?

Response: Thank you for your comment on the structure of our paper. We realized that the previous version lacks a high-level introduction of SCADIE’s design and applicability at the beginning due to Genome Biology’s requirement of putting Methods to the end. We have thus re-written the introduction section and provided a concise but comprehensive introduction at the end of this section so that the setup of our method can be better understood, we also modified Figure 1 to better inform this information. (Page 3 line 5-13, 21-29)

Regarding the single cell questions: 1.In the case where scRNA-seq is available for both case and control samples, cell type-specific differential expression analysis can directly be performed. By design SCADIE is still primarily for bulk RNA-Seq data.

2.If we would like to use case-control scRNA-Seq only for reference, the recommended way is to (a) generate signature matrices for the case and control groups, respectively, (b) perform deconvolution to obtain initial H1, H2, then (c) input bulk gene expression along with H1, H2 into the model.

In this revision, we have made it clear in our manuscript that users can either input bulk gene expression with Hs, or with a common signature matrix.

4) A lot of the short paragraphs contain a sentence of two. For example, the section "DEG identification under poor initialization and limited sample size" on pages 13-14. Also, the paper contains several bulletin points and lists within the main manuscript.

This is rarely seen in published journal articles.

Response: Thank you for your comment on our writing. We have reviewed and merged all the coherent short paragraphs. As for the bullet points, we have paraphrased all the bullet points into paragraphs except two indexings in section “Overview of SCADIE” and “Warm-up run and weight matrix derivation”, where we need to walk through the algorithm step by step.

5) On page 5, some high-level introduction to the SCAD function is needed - this is the key methodological contribution of this paper, and SCAD is also in the title. So some background is certainly needed.

Response: Thank you for this important comment. In the “Background” section of the revised manuscript, we have included the definition and the high-level introduction to the SCAD function. Hope that this update provides a better explanation of the SCAD method.

6) For the section "Robustness with respect to Zeta_n", Zeta_n is not even introduced in the main manuscript, and this can be confusing to the readers.

Response: Thank you for pointing this out. In the “Background” section of the revised manuscript, we have introduced Zeta_n when presenting the SCAD function (Page 2: line 35-44, page 3: line 1-4).

I am concerned with the false positives and false negatives of the methods. For the example shown in Figure 5, what's the difference between the two columns in table f, "the percentages that were significant from SCADIE output" and "the percentages that were correctly significant from SCADIE?" The difference is quite dramatic - are they called to be in different directions by SCADIE vs by single-cell case-control data? Also, for the thousands of the additional DE genes called by SCADIE, the authors picked the top 42 DE genes unique to SCADIE, and identified 19 to be associated with COPD and 33 to have the same directional change. What about the rest of the genes? A geneset enrichment analysis on the genes in the last column of table f? Similarly, for the boxplot in Figure 8, the boxplot of known DEGs was only marginally significant for the known up-regulated genes, but not for the down-regulated genes. Are these false negatives?

Response: Thank you for these helpful comments, we have included several additional analyses and made changes to address these issues, specifically:

1.We have redesigned the table in Figure 5 to only include the most important metrics, and also added some new benchmarking results.

Regarding the meanings of those columns in the previous table (shown below, not the same as the table in the manuscript): a) "the percentages that were significant from SCADIE output" represents the percentage of single cell DEGs that were identified as DEG from SCADIE, no matter if they were identified as DEGs of the correct direction or the opposite direction. And b) "the percentages that were correctly significant from SCADIE” represents the percentage of single cell DEGs that were identified as having the same direction DEG from SCADIE. The differences between these two columns are the proportions of genes that were identified as DEG in one direction from single cell, but the opposite direction from SCADIE.

To better illustrate their meanings, we provide the table below that also shows the number of genes in each category. Note that the numbers in the “#SC DEGs that were identified as significant DEGs of the opposite directions” column and the numbers in the “#SC DEGs that were identified as significant DEGs of the correct directions” column sum to the numbers in the “#SC DEGs that were identified as significant DEGs from SCAIDE” column. The percentages in the corresponding columns should match them in the above table.

Cell#DEGs from single cell#SC DEGs that were identified as significant DEGs from SCAIDE (percentages)#SC DEGs that were identified as significant DEGs of the opposite directions#SC DEGs that were identified as significant DEGs of the correct directions (percentages)

Stromal437209 (47.8%)73136 (31.1%)

Myeloid21683 (38.4%)1271 (32.9%)

Lymphoid7524 (32%)816 (21.3%)

Epithelial42853 (12.4%)1340 (9.3%)

Endothelial293106 (36.1%)2680 (27.3%)

We can see that 3-15% of DEGs that ended up being DEG of the opposite directions, or false positives.

Although there is low consistency between SCADIE and single cell results, we demonstrated through an additional benchmarking analysis that this is more likely due to the intrinsic differences, which can be substantial, between the bulk and single cell datasets.

2.We have also included a GSEA analysis and showed that the top pathways from SCADIE and single cell overlap more than that expected by chance (Page 9: line 20- 28, supplementary figure S5).

3.Per your suggestion, we realized that the boxplot might not be an appropriate comparison to make and thus have removed it. As for your questions on what were presented in the boxplot, those were not false positives. The boxplot showed the list of known DEGs’ estimated expression levels from SCADIE and CIBERSORT, however, since those genes had distinct expression levels in the first place, we realized that it does not add to the manuscript to compare their estimated expression levels.

Since SCADIE adopts a SCAD-based function as well as NNLS during the iterations, the computational efficiency needs to be assessed. Is it even feasible to be run on the whole transcriptome?

Response: Thank you for this valuable comment. The answer to your question is yes, in fact, all the SCADIE analyses were performed on whole transcriptome throughout this paper.

To provide a more detailed comparison for computational efficiency, in the Supplementary Section S3.3 of the revised manuscript, we have included computational time of SCADIE using simulation study in which the number of genes was varied from 500 to 10,000. We observe that the proposed algorithm only took a few minutes even when the number of genes was 10,000. This suggests that the proposed algorithm is feasible to be run on the whole transcriptome. Please see Supplementary Section S3.3 for the detailed results.

Similar to csSAM, does SCADIE perform a joint test between W and H? Also, an omnibus test across cell types?

Response: To our understanding, the interpretation of csSAM results requires comparing H and W separately, as they have different meanings (proportions vs gene expression). But in terms of functionality, SCADIE can provide all the equivalent information or testing that csSAM provides.

For the objective functions shown on pages 17 and 18, the first two terms are given equal weights, but their dimensions can differ. A similar framework (albeit on different types of data)

https://nam12.safelinks.protection.outlook.com/?url=https%3A%2F%2Fdoi.org%2F10.1

073%2Fpnas.1805681115&amp;data=04%7C01%7Chongyu.zhao%40yale.edu%7C54

296a5e60a24b00b7d508d9785d9c67%7Cdd8cbebb21394df8b4114e3e87abeb5c%7C

0%7C0%7C637673165725488032%7CUnknown%7CTWFpbGZsb3d8eyJWIjoiMC4wL

jAwMDAiLCJQIjoiV2luMzIiLCJBTiI6Ik1haWwiLCJXVCI6Mn0%3D%7C1000&amp;sdat

a=VFaR%2BCg4sNzTJaC4KtbX1xM0vQSeQ%2F9KtYy%2BwgGV7lQ%3D&amp;rese

rved=0 gives another tuning parameter to the second term.

Response: Thank you for your comment. As you mentioned, if two dimensions n1 and n2, are very different, then it may need different weights on the first two terms of the objective function. In Supplementary Section S3.5 of the revised manuscript, we have included simulation analysis by varying the two dimensions. Specifically, we varied the ratio of n2/n1 from 0.02 to 50. Note that the ratios of the two sample sizes in our real data analyses were between 0.1 and 10. It can be seen that the proposed algorithm with the equal weight on the first two terms still provided accurate results when the ratios were between 0.1 and 10. Please see Supplementary Section S3.5 for details. However, to cover the cases when the two sample sizes are very different, we proposed the weighted SCADIE by imposing weights w1=n2/n1, w2=1 for the first two terms. This is motivated by the fact that the first two terms can be understood as empirical risks, by averaging the loss function on the samples. According to the results in Supplementary Section S3.5 of the revised manuscript, weighted SCADIE outperformed SCADIE when the ratio n2/n1 was very small or large. In the updated SCADIE package in R, we incorporate this update by giving users an option to impose different weights on the first two terms depending on the two dimensions, n1 and n2. We totally agree that imposing another tuning parameter to the second term can improve the performances of SCADIE, but it will take additional time to tune the additional tuning parameter. We leave this issue for future research.

We hope that these updates will be received favorably.

Reviewer #2: The authors proposed the SCADIE approach, an iterative algorithm that can simultaneously estimate cell type-specific gene expression profiles and cell type proportions, and perform cell type-specific differential expression analysis at the group level. The work is interesting with a few novel ideas particularly the Lasso-type joint learning strategy, and may complement many existing supervised deconvolution methods. However, there are some major concerns that need to be carefully addressed and some conclusions may be adjusted. My specific critiques are:

1) Methods for computational deconvolution may be divided into supervised and unsupervised categories with a few exceptions of semi-supervised or sample-level approaches. To my understanding, SCADIE is a supervised population-level deconvolution method with unique focus on two group differential analysis. This nature should be clarified clearly.

Response: Thank you for your insightful comment. We have re-written most of the introduction part, and have emphasized this nature in the 6th and 7th paragraphs in the “Background” section (Page 2: 25-34; Page 3: 1-13).

2) The inputs required and assumptions made by SCADIE should be explicitly explained, e.g., proportion matrix or common signature matrix, and assumed known yet common number of cell types within/across groups. It is unclear whether the number and identify of cell types across two groups are assumed to be known and the same. For some disease lesions, some cell types are significantly altered, for example, in tumor or anthracnosis, involving novel cell subtypes that have no reference to be used (e.g. due to novel mutations). The authors should discuss these issues and clarify the applicability of SCADIE in a broad context.

Response: Thank you for your comment on the structure of our paper. Our previous manuscript lacks a high-level introduction of SCADIE’s design and applicability at the beginning due to Genome Biology’s requirement on putting Methods to the end. We have re-written the Introduction (“Background”) and the “Results – Overview of SCADIE” sections, where we provide a concise but comprehensive introduction of the model assumptions and applicability so that the setup of our method can be better understood.

3) In terms of biological diversity, some cell types are made up of complex mixtures rather than simple or known cell types. Microenvironment, cell-cell interaction, and developmental maturity are a few examples of situations when the subtypes are difficult to define. While cells can participate in various biological functions, we may need to acknowledge that one cell may fit into different subtype categories based on different classification methods, and they are all valid subtypes. There are several unsupervised deconvolution methods which have been proposed and some are readily accessible thus should not be completely disregarded (Hart, Sheftel et al. 2015, Chen, Wu et al. 2020). The authors may want to give a brief overview on this alternative.

Response: Thank you for your insightful comment. We were aware of the genre of unsupervised and semi-supervised deconvolution methods. In the early development stage of SCADIE, we tried to incorporate an un-supervised method linseed (https://www.nature.com/articles/s41467-019-09990-5) into our framework. However, we ended up not adopting linseed because: 1) we found that this unsupervised method performed poorly when the number of cell types exceeded 5. Another issue with the unsupervised method is that we need to manually annotate the outcome cell types, which sometimes is not possible as those cell types’ expression profiles do not match any of our reference expression profiles for known cell types.

We agree that cell type definition is highly versatile and dynamic depending on the analytical context. In this regard, we have made it clear in this revision (2nd paragraph in the "Results – Overview of SCADIE” section) that SCADIE is only applicable to cases where the compositional cell types are the same between groups.

4) Some statements are either unclear or inaccurate and should be corrected, e.g., "It is easy to see that directly estimating W and H without any constraint is not an identifiable problem.", "most deconvolution methods only focus on a subset of cell type- specific genes (i.e. signature genes), while all the genes in the genome are of interests in DEG analyses." Does 'simultaneously/directly' imply supervised or unsupervised approach?

Response: Thank you for these suggestions, we have re-written the whole paragraph in a clearer way. In terms of the meaning of “simultaneous”, we use it to refer to the fact that SCADIE can estimate W and H together, unlike conventional deconvolution methods which mainly focus on estimating H. We also explicitly explain this difference in the updated version ("Background” section, paragraphs 4-7 or page 2 line 6 to page 3 line 5).

Many deconvolution methods are applicable to deconvoluting all genes once the model is estimated

Response: Thank you for your insightful comment. In our revision, we have thus included benchmarking with two additional methods: MuSic, which utilizes all genes for deconvolution, and TOAST, which performs similar task as SCADIE. Based on our comparison results, SCADIE maintains competitive performance over these two methods.

5) The selection of K-L divergence to measure H accuracy needs some justification. Will such a measure be dominated by the abundant cell types?

Response: Thank you for pointing this out, and one other reviewer has brought up the same similar question.

The reason why we used K-L divergence to measure the dissimilarity of columns of matrices H and \hat{H} is that these matrices are proportion matrices, i.e., sum of each column is equal to one, so we can view each column as a discrete probability distribution. Although K-L divergence measures the loss well when the distribution is approximated by another distribution, we agree that it can be dominated by the abundant cell types because it considers log transformation of the ratio of two distributions. In the revised manuscript, we also report RMSE to measure the similarity of columns of matrices H and \hat{H}. We observe that the results between K-L divergence and RMSE are overall highly consistent. In the revised manuscript, we have also included benchmarking results using RMSE as metric (see Supplementary Figure S3).

6) In Figure 1, it is unclear whether cell type proportion matrix indicated in the text or signature matrix shown in the figure is required by SCADIE. Please clarify.

Response: Thank you for pointing this out. We apologize for the inconsistency between the main text, Figure 1 description and Figure 1 in the previous manuscript. In the revised manuscript, we have re-written the introduction paragraph, Figure 1 description, and modified Figure 1 to make it clear that SCADIE can either take bulk matrix + shared signature matrix, or bulk matrices + initial Hs as input.

7) Please clarify 'point estimates', 'entry-wise', etc.

Response: We have re-written the description for Figure 1c using pre-defined specific notations to describe and refer to these vague terms (please refer to the description for Figure 1).

8) Please explain what 'ROC-like curve' is and how it was generated. It is unclear why the ROC-like curves of the two peer methods are short and 'separated'. How were the ground truth DEGs specified?

Response: Thank you for your questions and suggestion. We have replaced the term “ROC-like curve” with more clear and comprehensible expression (e.g., True Positive Rates vs False Positive Rates plot) in the “SCADIE can better identify DEGs” section. We also specified that due to the simulated nature of these datasets, the DEG status was already known. The reason why some methods’ curves are short and separated is because their output DEG p-values are polarized, that is, some genes’ p-values are extremely small while others are quite large. This makes the result insensitive to p-value cutoffs.

9) The statement 'It should be noted that since CIBERSORTx' high resolution mode is only able to recover a small fraction of Ws, its overall sensitivity is limited.' Needs to be explained.

Response: Thank you for your suggestion. We have elaborated this in more details in the revised manuscript (“SCADIE can better identify DEG” section, paragraph 4), specifically, since CIBERSORTx’s high resolution mode is designed for imputing a relatively small number of genes, many genes had missing outcomes when we tried to impute for whole transcriptome, and this led to the small true positive and small false positive rates.

10) Regarding the advantages of SCADIE (actually two aspects), the statement was not clear and should be revisited. Also, the text 'In terms of functionality, SCADIE is the first computational method that can estimate and perform cell type-specific differential expression analysis at whole transcriptome level, provide matched cell type proportion estimates, while working with multiple types of expression data.' is an overstatement and should be adjusted or specified.

Response: Thank you for your suggestion, we have revised the overstatement in abstract, and provided more clear statements on the advantages of SCADIE in both abstract and introduction parts.

Hart, Y., H. Sheftel, J. Hausser, P. Szekely, N. B. Ben-Moshe, Y. Korem, A. Tendler, A. E. Mayo and U. Alon (2015). "Inferring biological tasks using Pareto analysis of high- dimensional data." Nat Methods 12(3): 233-235.

Chen, L., C. T. Wu, N. Wang, D. M. Herrington, R. Clarke and Y. Wang (2020). "debCAM: a bioconductor R package for fully unsupervised deconvolution of complex tissues." Bioinformatics 36(12): 3927-3929.

Reviewer #3: Genome Biology Simultaneous estimation of cell type proportions and cell type-specific gene expressions using SCAD-based iterative estimating procedure The research tries to bring an iterative algorithm to simultaneously address the estimation of cell-type proportions and cell type-specific gene expression in bulk sequencing data. Many existing deconvolution methods focused only on the cell type proportion estimation with the assumption of a fixed cell type-specific expression. Many differential expression genes analysis methods raised DEGs without the elimination of effects from diversified cell-type proportions. SCADIE, however, updates the two estimations together, offers more confidence on group-specific DEGs analysis. Also, compared to the existed deconvolution method, like CIBERSORTx, instead of working on a small signature matrix, SCADIE can work at the whole transcriptome level, thus offering more potential DEG candidates. SCADIE takes the combination of iterative NNLS optimization algorithms, SCAD-penalty, and jackknife method to carry out the whole estimation procedures. The highlighted part of the method lies in the SCAD penalty between cell type-specific expressions from two groups. Unlike the traditional penalization methods L1 or L2 penalties, the SCAD-penalty can set bounded regularization for the difference within a small range and at the same time penalize less on the large difference compared to L1/L2. This theoretically can help to regularize on the dissimilarity for cell type-specific expression in the two groups while at the same time maintain the true differences in the two groups. Thus, it carries out similarly to the practical cases. The paper is well written. The results and methods are quite clear and easy to understand. The mathematical part for the methods is also well-calibrated. The author also offers very detailed comparisons on a group of datasets including simulation, pseudo-bulk, and real bulk samples). However, when it comes to the results part, some of the results may be less convincing to support their claimed advantages compared to the existed methods.

Response: Thank you for your overall positive comments and detailed suggestions that we address in the following.

Few comments for the results and methods in the paper:

Results: Cell-type proportions estimations: For cell-type proportions estimations, SCADIE benchmarked against DWLS, CIBERSORTx, and naïve NNLS. Given the pseudo-bulk dataset has the single-cell reference data, and MuSiC is also a state-of- art method, a comparison including MuSiC would be more convincing. (Page 3, line 47)

Response: Thank you for your advice. We have included MuSiC in our benchmarking in this revision. MuSiC outperforms SCADIE in a pure single cell setting.

The evaluated metric for cell-type proportions is K-L divergence in this manuscript. For benchmark papers in the deconvolution area, the standard and stringent metrics are mostly correlation, mean absolute deviance, or RMSE. (Page 3, line 56)

Response: Thank you for your advice, and a similar concern was raised by another reviewer. In this revision we have included additional benchmarking results using RMSE as metric (see Supplementary Figure S3). The results between K-L divergence and RMSE are overall highly consistent. We believe cross-checking between two metrics should be sufficient and thus did not benchmark under MAD or correlation. In supplementary figure S3a.

The performance of SCADIE is not significantly better compared to the other methods. Raising the question of improvement on the accuracy of H (cell type proportions) over iterative estimations.

Response: Thank you for pointing out this issue. As we mentioned in the previous manuscript, our primary concern for SCADIE during the development and evaluation of SCADIE was that its iterative procedure might worsen the proportion estimates due to error accumulation and whole W based NNLS. To this end, the primary goal for benchmarking cell type proportion is to ensure that the H estimate will not be affected during iteration to allow us to estimate differentially expressed genes for each cell type. Our results suggest that the overall performance of SCADIE is satisfactory and better than other methods in most situations, which met our expectation.

SCAIE can better identify DEGs: For DEG analysis, before simultaneous estimation, we can estimate W (cell type-specific expression) from the proportions calculated from devolution tools first, and then carried out DEG analysis for two groups.

Here in Figure2, only NNLS, csSAM, CIBERSORTx are included. And csSAM is published for microarray deconvolution ten years ago. Including the W estimated from MuSiC etc. and include their DEG analysis would be more convincing. (Page 5, line 5)

Response: Thank you very much for this suggestion. After carefully reviewing MuSic’s model design, we believe it is currently not possible to perform DEG analysis based on its output because MuSiC assigns different weights to W’s rows in each run, and its output only contains weights but no the actual W matrix, and these weights are usually different between runs, making the weighted Ws not comparable for DEG analysis. However, we did include MuSiC in cell type proportion estimate benchmarking in our revision.

In Figure2, the DEG identification performance of SCADIE is not that significant as the author has claimed. In most cases, the performance overlapped with NNLS, while in the cases where the FPR decreases the TPR also decreases (except for one case, Mouse ISC Pseudo Bulk1 -Up DEGs). This can raise from the decrease in the number of DEGs detected. (Page 5, line 60; Page 6, line 8)

Response: Thank you for your comment on the similarity between SCADIE and NNLS outcomes in some cases. The reason why this happens is because the SCADIE iterative procedure begins with a few “warm-up” iterations using NNLS. In the case where the intermediate Ws and Hs from “warm-up” runs are close to convergence, the subsequent SCADIE updates do not change the results substantially, among the three datasets in Figure 2, example cases for this scenario are the “Simulation data” and “Mouse ISC Pseudo Bulk2”. While in case like “Mouse ISC Pseudo Bulk1”, the SCADIE procedure can substantially improve the intermediate results. Given the polarizing nature of SCAD penalty, the results from SCADIE in “Simulation data” and “Mouse ISC Pseudo Bulk2” are more spread-out than it is from NNLS. Here we take Figure 1 – Simulation Data Up DEG case as an example. In this case we can see that when p-cutoff is large, NNLS has higher true positive and false positive rates compared to SCADIE. However, the table below shows that in those cases, NNLS’s marginal incremental true positives are at very high cost of false positives. This suggests that it is still useful to incorporate the SCAD penalty. Besides, we also provide the option to run NNLS in our R package (as stated in section “SCADIE can better identify DEGs” paragraph 2) if the user thinks it is better to proceed that way.

Partial outcome for DEG Simulation Data Up DEG

p-cutoffNNLS True PositiveNNLS False PositiveSCADIE True PositiveSCADIE False

Positive

0.373.0%5.0%73.2%3.4%

0.477.1%9.4%75.2%6.6%

0.580.6%16.4%77.8%12.1%

Improve the estimates from other methods In Figure3, the improvement for accuracy with the iterative procedure is not that clear. For many cases, with a decrease or increase in both FPR and TPR.

Response: Thank you for raising this issue. For similar reasons as above, we think SCADIE still has its merits, specifically: in the Simulation Data case, SCADIE outperformed both competing methods in both true positive and false positive rates. In the Pseudo bulk data1 case, SCADIE achieved better true positive-false positive balance. In the Pseudo bulk data2 case, SCADIE performed worse in the up DEG case, but in the down DEG case, it achieved much better true positive rate at reasonable cost of false positive.

SCADIE's performance on real data (COPD) Figure4 offers a clear cell-type proportions structure. And carry out the large epithelial proportion variation for COPD groups, which is consistent well with the literature. In Figure5, 60% of single-cell identified DEGs showed just concordant directional changes from SCADIE output, and only 12-48% of single-cell identified DEGs were inferred to be DEGs by SCADIE. The percentages are not that strong. So, the claim of good performance might need support from the comparison with other methods. (Page 7, line 60)

Response: Thank you for pointing this out. We have added additional benchmarking along with other analyses for the COPD dataset. The results suggest that the low ratio was partially due to the substantial heterogeneity between the bulk and single cell COPD dataset. However, even in this case, SCADIE can still outperform a competing method by large margin.

Literature review to find the associations between SCADIE-unique genes and COPD better testify the signals detected from SCADIE. (Page 9, line 12)

SCADIE's performance on real data (AD) In Figure7, after the SCADIE procedure, the ratios of known DEGs that are estimated to have fold-changes in the opposite directions decreases. However, the p-values and fold-changes values are also sacrificed, with more DEGs fall into the insignificant regions. (Page 11, line 9) DEG identification under poor initialization and limited sample size The results may depend on the initial results of estimated cell-type proportions from other deconvolution methods, as well as the quality of the data. (Page 11, line 47) How to determine the maximum number of cell types the SCADIE can deal with is not mentioned. Should be discussed for the user to have a better idea about the cell type numbers they can apply with the given sample number. (Page 11, line 62) In Figure8, the SCADIE final W in 8a, though identified more up-regulated and down-regulated DEGs, but also more down-regulated DEGs detected as up-regulated. And the log2-expression fold change in 8b, though the p-values decrease compared to the CIBERSORTx, they are still insignificant, which proves again the SCADIE's estimation performance depends heavily on the correct initialization and enough sample size. (Page 12, line 47-54)

Response: Thank you for these insightful comments. We agree that the initialization quality and sample size can have substantial impacts on the outcome, and some comprehensive analyses are needed to provide practical guidelines. To this end, we have performed additional simulation analyses to comprehensively evaluate their impacts. Figure S6 shows that the DEG outcome with respect to initializations, and Supplementary Section S3.4 discusses the performances under different sample size to cell type ratios. Although it is not quite possible to evaluate initial H quality, but for sample size, we found out that SCADIE shows decent performance when the sample size is 1.5x the number of cell types. This explains why the performance in Figure 8 was poor (sample size/cell type ratio = 1.25). We also would like to note that similar methods like CIBERSORTx High Resolution Mode or TOAST usually require sample size 3x of cell types, so SCADIE still maintains an advantage here.

Methods: They build the whole algorithm with the assumption that most genes should have a proper dissimilarity level between the two groups of samples, which resembles many practical settings, also support the theoretical proposal for the selected penalty. (Page 2, line 46) The two highlighted novel strategies for SCADIE are: The selection of a SCAD-derivative based penalty function (Page 3, line 30) A parallel leave-one-out jackknife procedure to obtain the standard error for cell type- specific expression difference between the two groups. This can ease the further DEG analysis. (Page 3, line 37) Given DEG analysis is one of the key advantages the author tried to highlight in many sections. The hypothesis testing procedures might need a more detailed description in the manuscript. (Page 3, line 42).

Response: Thank you for your suggestion, we have included it as the step 5 in the “Overview of SCADIE” section.

The reasonable range is not clearly presented. (Page 6, line 60)

Response: Thank you for pointing this out for us, we have provided a more detailed range in the “Robustness with respect to $\zeta_{n}$” section.

The selected value for is not clearly discussed in the paper. (Page 15, line 28)

Response: Thank you for your comment. In the revised manuscript, we have specified the number of rounds we used in our analyses and pointed out that the number of warm-up runs can be changed in the “Warm-up run and weight matrix derivation” section.

Might change a to to keep consistency. (Page 16, line 18-22)

Response: Thank you for pointing out this notational inconsistency. In the revised manuscript, we have fixed “\alpha” to “a” in the SCAD function for consistency.

Given jackknife standard estimation is one of the key highlights in SCADIE, its advantage over bootstrap's sampling is not clearly presented. For the results provided in Supplementary section S3.3, these two methods have a high concordance. The potential singularity issue is not well explained and presented. (Page 16, line 61)

Response: Thank you for your comments. We would like to note that in general, the bootstrap is more computationally intensive than Jackknife (Sinharay, S. (2010)). Beside a potential singularity issue of Bootstrap, the main reasons that we prefer Jackknife are that Jackknife tends to perform better for confidence interval estimation for pairwise agreement measures and the Jackknife is more suitable for small data samples (Efron, B. (1982)). We have included this additional explanation in Supplementary Section S3.6 of the revised manuscript.

References

Efron, B. (1982) The Jackknife, the Bootstrap and Other Resampling Plans. CBMS- NSF Regional Conference Series in Applied Mathematics, Monograph 38, SIAM, Philadelphia.

Sinharay, S. (2010): Jackknife Methods. International Encyclopedia of Education (Third Edition), Elsevier

**2^nd^ round**

**Reviewer 1**

The authors have addressed my previous concerns.

**Reviewer 2**

1. The revised manuscript is much improved with satisfactory responses and revisions, while can be further improved.

2. It is still unclear how SCADIE obtains the number and types of various cell subpopulations present in a particular dataset?

3. It would be interesting to know the performance or robustness of SCADIE when the supervising information is less accurate or even incomplete with respect to a particular dataset. This information would be very useful to the users of SCADIE particularly when potential false discovery is a concern.

4. My understanding is that the fused-Lasso based re-estimation is indeed a very novel strategy and critical step in SCADIE that may significantly reduce FDR when detecting subtype-specific DEGs. If this is the case, I would suggest the authors to highlight this novelty and provide some results on sensitivity and specificity with and without this step if still possible.

5. I would suggest the authors to consider briefly mention about some unsupervised methods as alternatives, while I do agree with the authors that those methods may be imperfect.

**Reviewer 3**

All concerns are addressed.

**Authors’ response**

We would like to thank you again for your additional and very helpful comments as well as suggestions. Per your suggestions, we have made the following changes to make this manuscript more comprehensible:

More descriptions on the data analysis processes, particularly how the cell type categorizations were determined in each situation.

Extended our previous analyses to examine SCADIE’s performance more comprehensively with a focus on poor supervising information.

Clarified and emphasized the novelty of SCAD-penalty in false positive control through more comprehensive results.

Discussed SCADIE’s compatibility with unsupervised methods.

Please find below detailed point-by-point responses. We hope that these changes can address all your previous concerns, and please let us know if you have additional questions and comments.

Thank you all very much, and we look forward to hearing back from you!

Daiwei Tang, Seyoung Park, and Hongyu Zhao

Reviewer 2

It is still unclear how SCADIE obtains the number and types of various cell subpopulations present in a particular dataset?

Thank you for raising this question. The determination of cell type number and subpopulations depends on several factors:

The minimum granularity needed to answer the scientific question from deconvolution.

Differentiability among component cell types. As suggested by earlier research, including cell types with highly correlated expression profiles might introduce errors due to collinearity1.

Data availability: this is often the major bottleneck as the initial W or H matrix is usually available for a certain granularity. For example, cell type proportions estimated from experimental techniques like Immunohistochemistry can rarely cover more than five cell types.

The determination of cell types throughout this paper is a blend of the above considerations:

COPD dataset: the five cell types were determined according to the UMAP clustering results in the single cell paper we used for generating the signature matrix. Although the original paper clustered the single cell populations into 37 cell types, we decided to focus on the five major clusters because: 1) there are limited cell numbers in some clusters; and 2) correlated expression profiles of cell types within each cluster might introduce unwanted collinearity in W. We have included detailed description in the Real Data Processing and Analyses section on page 23 of the revised manuscript.

DLBLC dataset: the signature matrix used for this analysis was from a previous paper2 and the number of cell types used in our analysis was determined accordingly. We also added a short description on page 11 of the revised manuscript.

Alzheimer’s disease dataset: we considered three of the cell types for analysis due to the different cell type categorizations between the cell type proportion study3 and the DEG study4 we referred to. This reason is explained on page 12 in the revised manuscript.

CREBBP dataset: the choice of eight cell types in this analysis was out of three considerations: 1) the leave-one-out nature of Jackknife requires the number of cell types not exceeding #sample size-1, which is 9 in this case 2) we merged cell types into eight based on the biological question we were asking (DEGs in B cell) and their similarity (e.g. macrophage and monocyte were merged because their expression profiles are similar). A modified description on the above considerations can be found on page 13 and more elaborations on page 21 of the revised manuscript.

References:

Li, B., Liu, J.S. & Liu, X.S. Revisit linear regression-based deconvolution methods for tumor gene expression data. Genome Biol 18, 127 (2017). https://doi.org/10.1186/s13059-017-1256-5

Newman, A.M., Steen, C.B., Liu, C.L. et al. Determining cell type abundance and expression from bulk tissues with digital cytometry. Nat Biotechnol 37, 773–782 (2019). https://doi.org/10.1038/s41587-019-0114-2

Patrick E, Taga M, Ergun A, Ng B, Casazza W, Cimpean M, et al. (2020) Deconvolving the contributions of cell-type heterogeneity on cortical gene expression. PLoS Comput Biol 16(8): e1008120. https://doi.org/10.1371/journal.pcbi.1008120

Mathys, H., Davila-Velderrain, J., Peng, Z. et al. Single-cell transcriptomic analysis of Alzheimer’s disease. Nature 570, 332–337 (2019). https://doi.org/10.1038/s41586-019-1195-2

2. It would be interesting to know the performance or robustness of SCADIE when the supervising information is less accurate or even incomplete with respect to a particular dataset. This information would be very useful to the users of SCADIE particularly when potential false discovery is a concern.

Thank you for pointing this out. We agree that understanding SCADIE’s performance with inaccurate supervising information is indeed a key consideration given the fact that most real case supervising information is inaccurate. In the application of SCADIE, supervising information can be either the W (signature matrix) or H matrix. Since SCADIE supports different ways of initial deconvolution using signature matrix, it makes more sense to compare SCADIE’s performances under different initial H accuracy levels.

Due to the unknown ground truth in real data, we added simulation results to the DEG identification under poor initialization and limited sample size section (on page12) comparing DEG identification accuracy in a single dataset under different initial H’s accuracy levels. This part is coherent with the rest of the section where the performance was also indirectly evaluated in multiple real data sets. The takeaway is that H indeed affects DEG identification power, and in real applications users will at least need a check (based on their domain expertise) that the initial Hs are not too deviated from the underlying ground truth.

Regarding the case where the supervising information is missing (e.g. certain cell type’s proportion is missing), we do not think it would work on SCADIE as the underlying regression/NMF procedures would run into singularity issues. However, if the user opts to unsupervised deconvolution, their outputs can also be used for SCADIE analysis (without actually knowing which cell type is which).

3. My understanding is that the fused-Lasso based re-estimation is indeed a very novel strategy and critical step in SCADIE that may significantly reduce FDR when detecting subtype-specific DEGs. If this is the case, I would suggest the authors to highlight this novelty and provide some results on sensitivity and specificity with and without this step if still possible.

Thank you very much for this comment. The SCAD-based penalty term in our objective function was indeed introduced to reduce potential false positives and we consider it is one of the major novelties of this research (though we believe it is strictly not a fused LASSO).

We have included sensitivity, specificity, and positive predictive rate (PPV) results in supplementary figure S2 to better demonstrate its advantages. As can be seen in figure S2, introducing this penalty enables the algorithm to achieve high sensitivity level with better false positive control. It should be noted that due to the high #Non-DEGs/#DEGs ratio in figure S2, PPV is a more informative metric for false positive rate than specificity. Similar results can also be found in figure 2 when we compared specificity between SCADIE and NNLS.

To better emphasize its novelty, we have made changes in the following sections discussing its unique rationale and advantages:

Abstract (P1)

Section SCADIE can better identify DEGs (P7)

Section Rationale behind SCAD penalty (P16)

4. I would suggest the authors to consider briefly mention about some unsupervised methods as alternatives, while I do agree with the authors that those methods may be imperfect.

Thank you for your kind suggestion. We realized that although we had run into problems annotating the estimated cell types from unsupervised methods, these methods themselves are compatible with SCADIE’s scheme. Users can choose to initialize with unsupervised methods in special situations like lack of supervising information or cell type discovery. To this end, we have included description of the above in the Discussion section on page 15 of the revised manuscript.

**3^rd^ round**

The authors have addressed all my questions or concerns.
